# Supplementary material for: The causal effect of workplace violence on mental health and work-related outcomes: a cross-sectional study using propensity score matching
Source: PLoS One. 2025 May 7;20(5):e0321845. doi: 10.1371/journal.pone.0321845 (PMC12058019; doi:10.1371/journal.pone.0321845)
Supplement: S1 Appendix — (DOCX) [file pone.0321845.s001.docx]

Appendix 3. Effect sizes of violence in selected outcomes when stratified by population

| **Parameter** | **Group** | **RR (95% CI)** |
| --- | --- | --- |
| **Depressive symptoms** | Non PSM | 1.64 (1.47 to 1.83) |
| **Intention to quit** | Non PSM | 1.27 (1.14 to 1.41) |
| **Low professional fulfillment** | Non PSM | 1.45 (1.02 to 2.04) |
| **Depersonalization** | Non PSM | 1.57 (1.43 to 1.72) |
| **Emotional exhaustion** | Non PSM | 1.58 (1.42 to 1.77) |
| **Sleep problems** | Non PSM | 2.18 (1.71 to 2.79) |
| **No worklife balance** | Non PSM | 1.16 (1.07 to 1.27) |
| **Depressive symptoms** | Non PSM-nurse | 1.62 (1.41 to 1.87) |
| **Intention to quit** | Non PSM-nurse | 1.20 (1.03 to 1.40) |
| **Low professional fulfillment** | Non PSM-nurse | 2.07 (1.59 to 2.54) |
| **Depersonalization** | Non PSM-nurse | 1.61 (1.41 to 1.83) |
| **Emotional exhaustion** | Non PSM-nurse | 1.48 (1.26 to 1.74) |
| **Sleep problems** | Non PSM-nurse | 2.23 (1.59 to 3.14) |
| **No worklife balance** | Non PSM-nurse | 1.16 (1.02 to 1.33) |
| **Depressive symptoms** | Non PSM-doctor | 1.67 (1.41 to 1.97) |
| **Intention to quit** | Non PSM-doctor | 1.33 (1.14 to 1.54) |
| **Low professional fulfillment** | Non PSM-doctor | 1.04 (0.63 to 1.71) |
| **Depersonalization** | Non PSM-doctor | 1.53 (1.35 to 1.75) |
| **Emotional exhaustion** | Non PSM-doctor | 1.70 (1.46 to 1.97) |
| **Sleep problems** | Non PSM-doctor | 2.14 (1.51 to 3.07) |
| **No worklife balance** | Non PSM-doctor | 1.17 (1.04 to 1.33) |
| **Depressive symptoms** | PSM | 1.65 (1.47 to 1.86) |
| **Intention to quit** | PSM | 1.26 (1.13 to 1.41) |
| **Low professional fulfillment** | PSM | 1.45 (1.00 to 2.12) |
| **Depersonalization** | PSM | 1.58 (1.43 to 1.75) |
| **Emotional exhaustion** | PSM | 1.59 (1.41 to 1.79) |
| **Sleep problems** | PSM | 2.15 (1.65 to 2.81) |
| **No worklife balance** | PSM | 1.18 (1.07 to 1.30) |
| **Depressive symptoms** | PSM-nurse | 1.62 (1.37 to 1.91) |
| **Intention to quit** | PSM-nurse | 1.20 (1.01 to 1.43) |
| **Low professional fulfillment** | PSM-nurse | 1.92 (1.38 to 2.47) |
| **Depersonalization** | PSM-nurse | 1.67 (1.43 to 1.94) |
| **Emotional exhaustion** | PSM-nurse | 1.41 (1.18 to 1.70) |
| **Sleep problems** | PSM-nurse | 2.18 (1.48 to 3.28) |
| **No worklife balance** | PSM-nurse | 1.16 (1.00 to 1.34) |
| **Depressive symptoms** | PSM-doctor | 1.64 (1.39 to 1.95) |
| **Intention to quit** | PSM-doctor | 1.33 (1.14 to 1.55) |
| **Low professional fulfillment** | PSM-doctor | 1.00 (0.64 to 1.66) |
| **Depersonalization** | PSM-doctor | 1.52 (1.33 to 1.74) |
| **Emotional exhaustion** | PSM-doctor | 1.69 (1.45 to 1.97) |
| **Sleep problems** | PSM-doctor | 2.06 (1.45 to 2.97) |
| **No worklife balance** | PSM-doctor | 1.18 (1.04 to 1.34) |

PSM – Propensity Score Matching; non-PSM: non Propensity score matching.
