# Supplementary material for: The causal effect of workplace violence on mental health and work-related outcomes: a cross-sectional study using propensity score matching
Source: PLoS One. 2025 May 7;20(5):e0321845. doi: 10.1371/journal.pone.0321845 (PMC12058019; doi:10.1371/journal.pone.0321845)
Supplement: S2 Appendix — (DOCX) [file pone.0321845.s002.docx]

###############

## PACKAGES ##

##############

library(cobalt)

library("performance")

library(sandwich)

library(lmtest)

library(geepack)

library(MatchIt)

library(tableone)

library(ggplot2)

library(dplyr)

library(tidyr)

library(flexplot)

library(broom)

library(gt)

library(writexl)

#######################

### PREPARE DATASET ###

#######################

CURATED <- read_excel("CURATED.xlsx")

## Prepare your datasets

CURATED_NURSE <- CURATED

dimnames(CURATED_NURSE)

CURATED_NURSE$VIOLENCE <- ifelse(CURATED_NURSE$VIOLENCE == 4, 0, 1)

## analyze

dimnames(CURATED_NURSE)

##.TRANSFORM VARIALBES INTO DICHOTOMIC OUTCOMES

# SEX

table(CURATED_NURSE$SEX) # 0 FEMALE

# YEARS OF PRACTICE

table(CURATED_NURSE$YEARS_OF_PRACTICE) # 1 <10 YEARS, AS THIS IS A RISK FACTOR

CURATED_NURSE <- CURATED_NURSE %>%

mutate(YEARS_OF_PRACTICE = recode(YEARS_OF_PRACTICE,

`1` = "YOUNG",

`2` = "OLD"))

# LEVEL_OF_CARE

table(CURATED_NURSE$LEVEL_OF_CARE)

CURATED_NURSE <- CURATED_NURSE %>%

mutate(LEVEL_OF_CARE = recode(LEVEL_OF_CARE,

`1` = "PRIM",

`2` = "SEC",

`3` = "TERT"))

# MARITAL_STAT

table(CURATED_NURSE$MARITAL_STAT) #2 ERA CASADO, #1 ERA SOLO

CURATED_NURSE <- CURATED_NURSE %>%

mutate(MARITAL_STAT = recode(MARITAL_STAT,

`2` = "MARRIED",

`1` = "SINGLE"))

# DEPRESSION

CURATED_NURSE <- CURATED_NURSE %>%

mutate(DEP = case_when(

PHQ_2 == 2 ~ 0,

PHQ_2 >= 3 ~ 1,

TRUE ~ as.numeric(PHQ_2) # Si hay otros valores, los mantenemos iguales como numéricos

))

table(CURATED_NURSE$DEP)

# STABILITY

table(CURATED_NURSE$STABILITY) # 1 = NON JOB STABILITY (IN THE NEW)

CURATED_NURSE <- CURATED_NURSE %>%

mutate(STABILITY = recode(STABILITY,

`2` = "STAB",

`1` = "NON_STAB"))

# HOURS_PER_WEEK

table(CURATED_NURSE$HOURS_WEEKLY)

CURATED_NURSE <- CURATED_NURSE %>%

mutate(HOURS_WEEKLY = recode(HOURS_WEEKLY,

`4` = "MORE_80H",

`3` = "BET_60_79H",

`2` = "BET_40_59H",

`1` = "LESS_39H"))

# INSOMN

table(CURATED_NURSE$SLEEP_HOURS)

CURATED_NURSE <- CURATED_NURSE %>%

mutate(SLEEP_ADQ = ifelse(SLEEP_HOURS >= 7, 0, 1))

# HORAS

table(CURATED_NURSE$WORK_LIFE_BALANCE) # 4 AND 5 = OK | HENCE THIS WAS CATEGORIZED AS 0

CURATED_NURSE <- CURATED_NURSE %>%

mutate(WORK_LIFE_BALANCE_RECODED = ifelse(WORK_LIFE_BALANCE %in% c(4, 5), 0, 1))

table(CURATED_NURSE$FAMILY_SUPPORT)

CURATED_NURSE <- CURATED_NURSE %>%

mutate(FAMILY_SUPPORT = recode(FAMILY_SUPPORT,

`2` = "YES_FS",

`1` = "NON_FS"))

table(CURATED_NURSE$Highest_training)

CURATED_NURSE <- CURATED_NURSE %>%

mutate(HIGH_TRAIN = case_when(

Highest_training == 0 ~ "NADA",

Highest_training %in% 1:2 ~ "P_MASTER",

Highest_training == 3 ~ "MASTER",

Highest_training %in% 4:5 ~ "P_PHD",

Highest_training == 6 ~ "PHD",

TRUE ~ NA_character_ # Catch any unexpected values

))

table(CURATED_NURSE$HIGH_TRAIN)

CURATED_NURSE <- CURATED_NURSE %>%

mutate(SE = ifelse(BURN_D3 <= 34, 1, 0))

CURATED_NURSE <- CURATED_NURSE %>%

mutate(DEPERSO = ifelse(BURN_D2 >= 9, 1, 0))

CURATED_NURSE <- CURATED_NURSE %>%

mutate(EXHAUST = ifelse(BURN_D1 >= 26, 1, 0))

CURATED_NURSE <- CURATED_NURSE %>%

mutate(JENK = ifelse(INSOMN >= 12, 1, 0))

CURATED_NURSE <- CURATED_NURSE %>%

mutate(LEAVE_INTENTION = recode(LEAVE_INTENTION,

`2` = 1,

`1` = 0))

glimpse(CURATED_NURSE)

## Select the working dataset

all

all <- CURATED_NURSE %>%

select(

SEX, JOB, AGE, LEVEL_OF_CARE, MARITAL_STAT, FAMILY_SUPPORT,

LEAVE_INTENTION, STABILITY, YEARS_OF_PRACTICE, HOURS_WEEKLY,

PAID, PHYSICAL, AMENA, INSULT, SEXUAL_VIOL, VIOLENCE,

DEPARTMENT, DEP, WORK_LIFE_BALANCE_RECODED, HIGH_TRAIN,

SE, DEPERSO, EXHAUST, JENK, BURN_D1, BURN_D2, BURN_D3

)

all <- all %>%

mutate(HIGH_TRAIN = ifelse(HIGH_TRAIN == "NADA", "NON", "YES"))

all <- all %>%

mutate(DEPARTMENT = ifelse(DEPARTMENT == "LIMA", "LIMA", "PROVINCE"))

glimpse(all)

str(all)

all <- all %>%

mutate(HOURS_WEEKLY = ifelse(HOURS_WEEKLY == "LESS_39H", "NORM", "OVER"))

all <- all %>%

mutate(PAID = case_when(

PAID %in% c(1, 2) ~ "LESS_3K",

PAID %in% c(3, 4) ~ "FROM_3_7K",

PAID %in% c(5, 6) ~ "OVER_7K",

TRUE ~ as.character(PAID) # Default case, if any other values exist

))

# Check the result

table(all$PAID)

# SEX -

job_table <- all %>%

group_by(JOB) %>%

summarise(

perc = n() / 4951 *100,

n_viol = sum(VIOLENCE == "1"),

tot_viol = n(),

perc_viol = n_viol / tot_viol * 100,

viol_CI_lower = binom.test(n_viol, tot_viol)$conf.int[1] * 100,

viol_CI_upper = binom.test(n_viol, tot_viol)$conf.int[2] * 100,

n_viol_insult = sum(INSULT == "1"),

perc_viol_insult = n_viol_insult / tot_viol * 100,

viol_CI_lower_insult = binom.test(n_viol_insult, tot_viol)$conf.int[1] * 100,

viol_CI_upper_insult = binom.test(n_viol_insult, tot_viol)$conf.int[2] * 100,

n_viol_amena = sum(AMENA == "1"),

perc_viol_amena = n_viol_amena / tot_viol * 100,

viol_CI_lower_amena = binom.test(n_viol_amena, tot_viol)$conf.int[1] * 100,

viol_CI_upper_amena = binom.test(n_viol_amena, tot_viol)$conf.int[2] * 100,

n_viol_fis = sum(PHYSICAL == "1"),

perc_viol_fis = n_viol_fis / tot_viol * 100,

viol_CI_lower_fis = binom.test(n_viol_fis, tot_viol)$conf.int[1] * 100,

viol_CI_upper_fis = binom.test(n_viol_fis, tot_viol)$conf.int[2] * 100,

n_viol_sex = sum(SEXUAL_VIOL == "1"),

perc_viol_sex = n_viol_sex / tot_viol * 100,

viol_CI_lower_sex = binom.test(n_viol_sex, tot_viol)$conf.int[1] * 100,

viol_CI_upper_sex = binom.test(n_viol_sex, tot_viol)$conf.int[2] * 100

)

sex_table <- all %>%

group_by(SEX) %>%

summarise(

perc = n() / 4951 *100,

n_viol = sum(VIOLENCE == "1"),

tot_viol = n(),

perc_viol = n_viol / tot_viol * 100,

viol_CI_lower = binom.test(n_viol, tot_viol)$conf.int[1] * 100,

viol_CI_upper = binom.test(n_viol, tot_viol)$conf.int[2] * 100,

n_viol_insult = sum(INSULT == "1"),

perc_viol_insult = n_viol_insult / tot_viol * 100,

viol_CI_lower_insult = binom.test(n_viol_insult, tot_viol)$conf.int[1] * 100,

viol_CI_upper_insult = binom.test(n_viol_insult, tot_viol)$conf.int[2] * 100,

n_viol_amena = sum(AMENA == "1"),

perc_viol_amena = n_viol_amena / tot_viol * 100,

viol_CI_lower_amena = binom.test(n_viol_amena, tot_viol)$conf.int[1] * 100,

viol_CI_upper_amena = binom.test(n_viol_amena, tot_viol)$conf.int[2] * 100,

n_viol_fis = sum(PHYSICAL == "1"),

perc_viol_fis = n_viol_fis / tot_viol * 100,

viol_CI_lower_fis = binom.test(n_viol_fis, tot_viol)$conf.int[1] * 100,

viol_CI_upper_fis = binom.test(n_viol_fis, tot_viol)$conf.int[2] * 100,

n_viol_sex = sum(SEXUAL_VIOL == "1"),

perc_viol_sex = n_viol_sex / tot_viol * 100,

viol_CI_lower_sex = binom.test(n_viol_sex, tot_viol)$conf.int[1] * 100,

viol_CI_upper_sex = binom.test(n_viol_sex, tot_viol)$conf.int[2] * 100

)

higher_ed <- all %>%

group_by(HIGH_TRAIN) %>%

summarise(

perc = n() / 4951 *100,

n_viol = sum(VIOLENCE == "1"),

tot_viol = n(),

perc_viol = n_viol / tot_viol * 100,

viol_CI_lower = binom.test(n_viol, tot_viol)$conf.int[1] * 100,

viol_CI_upper = binom.test(n_viol, tot_viol)$conf.int[2] * 100,

n_viol_insult = sum(INSULT == "1"),

perc_viol_insult = n_viol_insult / tot_viol * 100,

viol_CI_lower_insult = binom.test(n_viol_insult, tot_viol)$conf.int[1] * 100,

viol_CI_upper_insult = binom.test(n_viol_insult, tot_viol)$conf.int[2] * 100,

n_viol_amena = sum(AMENA == "1"),

perc_viol_amena = n_viol_amena / tot_viol * 100,

viol_CI_lower_amena = binom.test(n_viol_amena, tot_viol)$conf.int[1] * 100,

viol_CI_upper_amena = binom.test(n_viol_amena, tot_viol)$conf.int[2] * 100,

n_viol_fis = sum(PHYSICAL == "1"),

perc_viol_fis = n_viol_fis / tot_viol * 100,

viol_CI_lower_fis = binom.test(n_viol_fis, tot_viol)$conf.int[1] * 100,

viol_CI_upper_fis = binom.test(n_viol_fis, tot_viol)$conf.int[2] * 100,

n_viol_sex = sum(SEXUAL_VIOL == "1"),

perc_viol_sex = n_viol_sex / tot_viol * 100,

viol_CI_lower_sex = binom.test(n_viol_sex, tot_viol)$conf.int[1] * 100,

viol_CI_upper_sex = binom.test(n_viol_sex, tot_viol)$conf.int[2] * 100

)

level_care <- all %>%

group_by(LEVEL_OF_CARE) %>%

summarise(

perc = n() / 4951 *100,

n_viol = sum(VIOLENCE == "1"),

tot_viol = n(),

perc_viol = n_viol / tot_viol * 100,

viol_CI_lower = binom.test(n_viol, tot_viol)$conf.int[1] * 100,

viol_CI_upper = binom.test(n_viol, tot_viol)$conf.int[2] * 100,

n_viol_insult = sum(INSULT == "1"),

perc_viol_insult = n_viol_insult / tot_viol * 100,

viol_CI_lower_insult = binom.test(n_viol_insult, tot_viol)$conf.int[1] * 100,

viol_CI_upper_insult = binom.test(n_viol_insult, tot_viol)$conf.int[2] * 100,

n_viol_amena = sum(AMENA == "1"),

perc_viol_amena = n_viol_amena / tot_viol * 100,

viol_CI_lower_amena = binom.test(n_viol_amena, tot_viol)$conf.int[1] * 100,

viol_CI_upper_amena = binom.test(n_viol_amena, tot_viol)$conf.int[2] * 100,

n_viol_fis = sum(PHYSICAL == "1"),

perc_viol_fis = n_viol_fis / tot_viol * 100,

viol_CI_lower_fis = binom.test(n_viol_fis, tot_viol)$conf.int[1] * 100,

viol_CI_upper_fis = binom.test(n_viol_fis, tot_viol)$conf.int[2] * 100,

n_viol_sex = sum(SEXUAL_VIOL == "1"),

perc_viol_sex = n_viol_sex / tot_viol * 100,

viol_CI_lower_sex = binom.test(n_viol_sex, tot_viol)$conf.int[1] * 100,

viol_CI_upper_sex = binom.test(n_viol_sex, tot_viol)$conf.int[2] * 100

)

mar_stat <- all %>%

group_by(MARITAL_STAT) %>%

summarise(

tot_viol = n(),

perc = n() / 4951 *100,

n_viol = sum(VIOLENCE == "1"),

perc_viol = n_viol / tot_viol * 100,

viol_CI_lower = binom.test(n_viol, tot_viol)$conf.int[1] * 100,

viol_CI_upper = binom.test(n_viol, tot_viol)$conf.int[2] * 100,

n_viol_insult = sum(INSULT == "1"),

perc_viol_insult = n_viol_insult / tot_viol * 100,

viol_CI_lower_insult = binom.test(n_viol_insult, tot_viol)$conf.int[1] * 100,

viol_CI_upper_insult = binom.test(n_viol_insult, tot_viol)$conf.int[2] * 100,

n_viol_amena = sum(AMENA == "1"),

perc_viol_amena = n_viol_amena / tot_viol * 100,

viol_CI_lower_amena = binom.test(n_viol_amena, tot_viol)$conf.int[1] * 100,

viol_CI_upper_amena = binom.test(n_viol_amena, tot_viol)$conf.int[2] * 100,

n_viol_fis = sum(PHYSICAL == "1"),

perc_viol_fis = n_viol_fis / tot_viol * 100,

viol_CI_lower_fis = binom.test(n_viol_fis, tot_viol)$conf.int[1] * 100,

viol_CI_upper_fis = binom.test(n_viol_fis, tot_viol)$conf.int[2] * 100,

n_viol_sex = sum(SEXUAL_VIOL == "1"),

perc_viol_sex = n_viol_sex / tot_viol * 100,

viol_CI_lower_sex = binom.test(n_viol_sex, tot_viol)$conf.int[1] * 100,

viol_CI_upper_sex = binom.test(n_viol_sex, tot_viol)$conf.int[2] * 100

)

fam_sup <- all %>%

group_by(FAMILY_SUPPORT) %>%

summarise(

tot_viol = n(),

perc = n() / 4951 *100,

n_viol = sum(VIOLENCE == "1"),

perc_viol = n_viol / tot_viol * 100,

viol_CI_lower = binom.test(n_viol, tot_viol)$conf.int[1] * 100,

viol_CI_upper = binom.test(n_viol, tot_viol)$conf.int[2] * 100,

n_viol_insult = sum(INSULT == "1"),

perc_viol_insult = n_viol_insult / tot_viol * 100,

viol_CI_lower_insult = binom.test(n_viol_insult, tot_viol)$conf.int[1] * 100,

viol_CI_upper_insult = binom.test(n_viol_insult, tot_viol)$conf.int[2] * 100,

n_viol_amena = sum(AMENA == "1"),

perc_viol_amena = n_viol_amena / tot_viol * 100,

viol_CI_lower_amena = binom.test(n_viol_amena, tot_viol)$conf.int[1] * 100,

viol_CI_upper_amena = binom.test(n_viol_amena, tot_viol)$conf.int[2] * 100,

n_viol_fis = sum(PHYSICAL == "1"),

perc_viol_fis = n_viol_fis / tot_viol * 100,

viol_CI_lower_fis = binom.test(n_viol_fis, tot_viol)$conf.int[1] * 100,

viol_CI_upper_fis = binom.test(n_viol_fis, tot_viol)$conf.int[2] * 100,

n_viol_sex = sum(SEXUAL_VIOL == "1"),

perc_viol_sex = n_viol_sex / tot_viol * 100,

viol_CI_lower_sex = binom.test(n_viol_sex, tot_viol)$conf.int[1] * 100,

viol_CI_upper_sex = binom.test(n_viol_sex, tot_viol)$conf.int[2] * 100

)

contract_stab <- all %>%

group_by(STABILITY) %>%

summarise(

tot_viol = n(),

perc = n() / 4951 *100,

n_viol = sum(VIOLENCE == "1"),

perc_viol = n_viol / tot_viol * 100,

viol_CI_lower = binom.test(n_viol, tot_viol)$conf.int[1] * 100,

viol_CI_upper = binom.test(n_viol, tot_viol)$conf.int[2] * 100,

n_viol_insult = sum(INSULT == "1"),

perc_viol_insult = n_viol_insult / tot_viol * 100,

viol_CI_lower_insult = binom.test(n_viol_insult, tot_viol)$conf.int[1] * 100,

viol_CI_upper_insult = binom.test(n_viol_insult, tot_viol)$conf.int[2] * 100,

n_viol_amena = sum(AMENA == "1"),

perc_viol_amena = n_viol_amena / tot_viol * 100,

viol_CI_lower_amena = binom.test(n_viol_amena, tot_viol)$conf.int[1] * 100,

viol_CI_upper_amena = binom.test(n_viol_amena, tot_viol)$conf.int[2] * 100,

n_viol_fis = sum(PHYSICAL == "1"),

perc_viol_fis = n_viol_fis / tot_viol * 100,

viol_CI_lower_fis = binom.test(n_viol_fis, tot_viol)$conf.int[1] * 100,

viol_CI_upper_fis = binom.test(n_viol_fis, tot_viol)$conf.int[2] * 100,

n_viol_sex = sum(SEXUAL_VIOL == "1"),

perc_viol_sex = n_viol_sex / tot_viol * 100,

viol_CI_lower_sex = binom.test(n_viol_sex, tot_viol)$conf.int[1] * 100,

viol_CI_upper_sex = binom.test(n_viol_sex, tot_viol)$conf.int[2] * 100

)

work_hours <- all %>%

group_by(HOURS_WEEKLY ) %>%

summarise(

tot_viol = n(),

perc = n() / 4951 *100,

n_viol = sum(VIOLENCE == "1"),

perc_viol = n_viol / tot_viol * 100,

viol_CI_lower = binom.test(n_viol, tot_viol)$conf.int[1] * 100,

viol_CI_upper = binom.test(n_viol, tot_viol)$conf.int[2] * 100,

n_viol_insult = sum(INSULT == "1"),

perc_viol_insult = n_viol_insult / tot_viol * 100,

viol_CI_lower_insult = binom.test(n_viol_insult, tot_viol)$conf.int[1] * 100,

viol_CI_upper_insult = binom.test(n_viol_insult, tot_viol)$conf.int[2] * 100,

n_viol_amena = sum(AMENA == "1"),

perc_viol_amena = n_viol_amena / tot_viol * 100,

viol_CI_lower_amena = binom.test(n_viol_amena, tot_viol)$conf.int[1] * 100,

viol_CI_upper_amena = binom.test(n_viol_amena, tot_viol)$conf.int[2] * 100,

n_viol_fis = sum(PHYSICAL == "1"),

perc_viol_fis = n_viol_fis / tot_viol * 100,

viol_CI_lower_fis = binom.test(n_viol_fis, tot_viol)$conf.int[1] * 100,

viol_CI_upper_fis = binom.test(n_viol_fis, tot_viol)$conf.int[2] * 100,

n_viol_sex = sum(SEXUAL_VIOL == "1"),

perc_viol_sex = n_viol_sex / tot_viol * 100,

viol_CI_lower_sex = binom.test(n_viol_sex, tot_viol)$conf.int[1] * 100,

viol_CI_upper_sex = binom.test(n_viol_sex, tot_viol)$conf.int[2] * 100

)

work_hours

income <- all %>%

group_by(PAID ) %>%

summarise(

tot_viol = n(),

perc = n() / 4951 *100,

n_viol = sum(VIOLENCE == "1"),

perc_viol = n_viol / tot_viol * 100,

viol_CI_lower = binom.test(n_viol, tot_viol)$conf.int[1] * 100,

viol_CI_upper = binom.test(n_viol, tot_viol)$conf.int[2] * 100,

n_viol_insult = sum(INSULT == "1"),

perc_viol_insult = n_viol_insult / tot_viol * 100,

viol_CI_lower_insult = binom.test(n_viol_insult, tot_viol)$conf.int[1] * 100,

viol_CI_upper_insult = binom.test(n_viol_insult, tot_viol)$conf.int[2] * 100,

n_viol_amena = sum(AMENA == "1"),

perc_viol_amena = n_viol_amena / tot_viol * 100,

viol_CI_lower_amena = binom.test(n_viol_amena, tot_viol)$conf.int[1] * 100,

viol_CI_upper_amena = binom.test(n_viol_amena, tot_viol)$conf.int[2] * 100,

n_viol_fis = sum(PHYSICAL == "1"),

perc_viol_fis = n_viol_fis / tot_viol * 100,

viol_CI_lower_fis = binom.test(n_viol_fis, tot_viol)$conf.int[1] * 100,

viol_CI_upper_fis = binom.test(n_viol_fis, tot_viol)$conf.int[2] * 100,

n_viol_sex = sum(SEXUAL_VIOL == "1"),

perc_viol_sex = n_viol_sex / tot_viol * 100,

viol_CI_lower_sex = binom.test(n_viol_sex, tot_viol)$conf.int[1] * 100,

viol_CI_upper_sex = binom.test(n_viol_sex, tot_viol)$conf.int[2] * 100

)

year_of_p <- all %>%

group_by(YEARS_OF_PRACTICE ) %>%

summarise(

tot_viol = n(),

perc = n() / 4951 *100,

n_viol = sum(VIOLENCE == "1"),

perc_viol = n_viol / tot_viol * 100,

viol_CI_lower = binom.test(n_viol, tot_viol)$conf.int[1] * 100,

viol_CI_upper = binom.test(n_viol, tot_viol)$conf.int[2] * 100,

n_viol_insult = sum(INSULT == "1"),

perc_viol_insult = n_viol_insult / tot_viol * 100,

viol_CI_lower_insult = binom.test(n_viol_insult, tot_viol)$conf.int[1] * 100,

viol_CI_upper_insult = binom.test(n_viol_insult, tot_viol)$conf.int[2] * 100,

n_viol_amena = sum(AMENA == "1"),

perc_viol_amena = n_viol_amena / tot_viol * 100,

viol_CI_lower_amena = binom.test(n_viol_amena, tot_viol)$conf.int[1] * 100,

viol_CI_upper_amena = binom.test(n_viol_amena, tot_viol)$conf.int[2] * 100,

n_viol_fis = sum(PHYSICAL == "1"),

perc_viol_fis = n_viol_fis / tot_viol * 100,

viol_CI_lower_fis = binom.test(n_viol_fis, tot_viol)$conf.int[1] * 100,

viol_CI_upper_fis = binom.test(n_viol_fis, tot_viol)$conf.int[2] * 100,

n_viol_sex = sum(SEXUAL_VIOL == "1"),

perc_viol_sex = n_viol_sex / tot_viol * 100,

viol_CI_lower_sex = binom.test(n_viol_sex, tot_viol)$conf.int[1] * 100,

viol_CI_upper_sex = binom.test(n_viol_sex, tot_viol)$conf.int[2] * 100

)

region <- all %>%

group_by(DEPARTMENT ) %>%

summarise(

tot_viol = n(),

perc = n() / 4951 *100,

n_viol = sum(VIOLENCE == "1"),

perc_viol = n_viol / tot_viol * 100,

viol_CI_lower = binom.test(n_viol, tot_viol)$conf.int[1] * 100,

viol_CI_upper = binom.test(n_viol, tot_viol)$conf.int[2] * 100,

n_viol_insult = sum(INSULT == "1"),

perc_viol_insult = n_viol_insult / tot_viol * 100,

viol_CI_lower_insult = binom.test(n_viol_insult, tot_viol)$conf.int[1] * 100,

viol_CI_upper_insult = binom.test(n_viol_insult, tot_viol)$conf.int[2] * 100,

n_viol_amena = sum(AMENA == "1"),

perc_viol_amena = n_viol_amena / tot_viol * 100,

viol_CI_lower_amena = binom.test(n_viol_amena, tot_viol)$conf.int[1] * 100,

viol_CI_upper_amena = binom.test(n_viol_amena, tot_viol)$conf.int[2] * 100,

n_viol_fis = sum(PHYSICAL == "1"),

perc_viol_fis = n_viol_fis / tot_viol * 100,

viol_CI_lower_fis = binom.test(n_viol_fis, tot_viol)$conf.int[1] * 100,

viol_CI_upper_fis = binom.test(n_viol_fis, tot_viol)$conf.int[2] * 100,

n_viol_sex = sum(SEXUAL_VIOL == "1"),

perc_viol_sex = n_viol_sex / tot_viol * 100,

viol_CI_lower_sex = binom.test(n_viol_sex, tot_viol)$conf.int[1] * 100,

viol_CI_upper_sex = binom.test(n_viol_sex, tot_viol)$conf.int[2] * 100

)

depresion <- all %>%

group_by(DEP) %>%

summarise(

tot_viol = n(),

perc = n() / 4951 *100,

n_viol = sum(VIOLENCE == "1"),

perc_viol = n_viol / tot_viol * 100,

viol_CI_lower = binom.test(n_viol, tot_viol)$conf.int[1] * 100,

viol_CI_upper = binom.test(n_viol, tot_viol)$conf.int[2] * 100,

n_viol_insult = sum(INSULT == "1"),

perc_viol_insult = n_viol_insult / tot_viol * 100,

viol_CI_lower_insult = binom.test(n_viol_insult, tot_viol)$conf.int[1] * 100,

viol_CI_upper_insult = binom.test(n_viol_insult, tot_viol)$conf.int[2] * 100,

n_viol_amena = sum(AMENA == "1"),

perc_viol_amena = n_viol_amena / tot_viol * 100,

viol_CI_lower_amena = binom.test(n_viol_amena, tot_viol)$conf.int[1] * 100,

viol_CI_upper_amena = binom.test(n_viol_amena, tot_viol)$conf.int[2] * 100,

n_viol_fis = sum(PHYSICAL == "1"),

perc_viol_fis = n_viol_fis / tot_viol * 100,

viol_CI_lower_fis = binom.test(n_viol_fis, tot_viol)$conf.int[1] * 100,

viol_CI_upper_fis = binom.test(n_viol_fis, tot_viol)$conf.int[2] * 100,

n_viol_sex = sum(SEXUAL_VIOL == "1"),

perc_viol_sex = n_viol_sex / tot_viol * 100,

viol_CI_lower_sex = binom.test(n_viol_sex, tot_viol)$conf.int[1] * 100,

viol_CI_upper_sex = binom.test(n_viol_sex, tot_viol)$conf.int[2] * 100

)

insomnia <- all %>%

group_by(JENK) %>%

summarise(

tot_viol = n(),

perc = n() / 4951 *100,

n_viol = sum(VIOLENCE == "1"),

perc_viol = n_viol / tot_viol * 100,

viol_CI_lower = binom.test(n_viol, tot_viol)$conf.int[1] * 100,

viol_CI_upper = binom.test(n_viol, tot_viol)$conf.int[2] * 100,

n_viol_insult = sum(INSULT == "1"),

perc_viol_insult = n_viol_insult / tot_viol * 100,

viol_CI_lower_insult = binom.test(n_viol_insult, tot_viol)$conf.int[1] * 100,

viol_CI_upper_insult = binom.test(n_viol_insult, tot_viol)$conf.int[2] * 100,

n_viol_amena = sum(AMENA == "1"),

perc_viol_amena = n_viol_amena / tot_viol * 100,

viol_CI_lower_amena = binom.test(n_viol_amena, tot_viol)$conf.int[1] * 100,

viol_CI_upper_amena = binom.test(n_viol_amena, tot_viol)$conf.int[2] * 100,

n_viol_fis = sum(PHYSICAL == "1"),

perc_viol_fis = n_viol_fis / tot_viol * 100,

viol_CI_lower_fis = binom.test(n_viol_fis, tot_viol)$conf.int[1] * 100,

viol_CI_upper_fis = binom.test(n_viol_fis, tot_viol)$conf.int[2] * 100,

n_viol_sex = sum(SEXUAL_VIOL == "1"),

perc_viol_sex = n_viol_sex / tot_viol * 100,

viol_CI_lower_sex = binom.test(n_viol_sex, tot_viol)$conf.int[1] * 100,

viol_CI_upper_sex = binom.test(n_viol_sex, tot_viol)$conf.int[2] * 100

)

int_leave <- all %>%

group_by(LEAVE_INTENTION) %>%

summarise(

tot_viol = n(),

perc = n() / 4951 *100,

n_viol = sum(VIOLENCE == "1"),

perc_viol = n_viol / tot_viol * 100,

viol_CI_lower = binom.test(n_viol, tot_viol)$conf.int[1] * 100,

viol_CI_upper = binom.test(n_viol, tot_viol)$conf.int[2] * 100,

n_viol_insult = sum(INSULT == "1"),

perc_viol_insult = n_viol_insult / tot_viol * 100,

viol_CI_lower_insult = binom.test(n_viol_insult, tot_viol)$conf.int[1] * 100,

viol_CI_upper_insult = binom.test(n_viol_insult, tot_viol)$conf.int[2] * 100,

n_viol_amena = sum(AMENA == "1"),

perc_viol_amena = n_viol_amena / tot_viol * 100,

viol_CI_lower_amena = binom.test(n_viol_amena, tot_viol)$conf.int[1] * 100,

viol_CI_upper_amena = binom.test(n_viol_amena, tot_viol)$conf.int[2] * 100,

n_viol_fis = sum(PHYSICAL == "1"),

perc_viol_fis = n_viol_fis / tot_viol * 100,

viol_CI_lower_fis = binom.test(n_viol_fis, tot_viol)$conf.int[1] * 100,

viol_CI_upper_fis = binom.test(n_viol_fis, tot_viol)$conf.int[2] * 100,

n_viol_sex = sum(SEXUAL_VIOL == "1"),

perc_viol_sex = n_viol_sex / tot_viol * 100,

viol_CI_lower_sex = binom.test(n_viol_sex, tot_viol)$conf.int[1] * 100,

viol_CI_upper_sex = binom.test(n_viol_sex, tot_viol)$conf.int[2] * 100

)

work_life <- all %>%

group_by(WORK_LIFE_BALANCE_RECODED) %>%

summarise(

tot_viol = n(),

perc = n() / 4951 *100,

n_viol = sum(VIOLENCE == "1"),

perc_viol = n_viol / tot_viol * 100,

viol_CI_lower = binom.test(n_viol, tot_viol)$conf.int[1] * 100,

viol_CI_upper = binom.test(n_viol, tot_viol)$conf.int[2] * 100,

n_viol_insult = sum(INSULT == "1"),

perc_viol_insult = n_viol_insult / tot_viol * 100,

viol_CI_lower_insult = binom.test(n_viol_insult, tot_viol)$conf.int[1] * 100,

viol_CI_upper_insult = binom.test(n_viol_insult, tot_viol)$conf.int[2] * 100,

n_viol_amena = sum(AMENA == "1"),

perc_viol_amena = n_viol_amena / tot_viol * 100,

viol_CI_lower_amena = binom.test(n_viol_amena, tot_viol)$conf.int[1] * 100,

viol_CI_upper_amena = binom.test(n_viol_amena, tot_viol)$conf.int[2] * 100,

n_viol_fis = sum(PHYSICAL == "1"),

perc_viol_fis = n_viol_fis / tot_viol * 100,

viol_CI_lower_fis = binom.test(n_viol_fis, tot_viol)$conf.int[1] * 100,

viol_CI_upper_fis = binom.test(n_viol_fis, tot_viol)$conf.int[2] * 100,

n_viol_sex = sum(SEXUAL_VIOL == "1"),

perc_viol_sex = n_viol_sex / tot_viol * 100,

viol_CI_lower_sex = binom.test(n_viol_sex, tot_viol)$conf.int[1] * 100,

viol_CI_upper_sex = binom.test(n_viol_sex, tot_viol)$conf.int[2] * 100

)

se <- all %>%

group_by(SE) %>%

summarise(

tot_viol = n(),

perc = n() / 4951 *100,

n_viol = sum(VIOLENCE == "1"),

perc_viol = n_viol / tot_viol * 100,

viol_CI_lower = binom.test(n_viol, tot_viol)$conf.int[1] * 100,

viol_CI_upper = binom.test(n_viol, tot_viol)$conf.int[2] * 100,

n_viol_insult = sum(INSULT == "1"),

perc_viol_insult = n_viol_insult / tot_viol * 100,

viol_CI_lower_insult = binom.test(n_viol_insult, tot_viol)$conf.int[1] * 100,

viol_CI_upper_insult = binom.test(n_viol_insult, tot_viol)$conf.int[2] * 100,

n_viol_amena = sum(AMENA == "1"),

perc_viol_amena = n_viol_amena / tot_viol * 100,

viol_CI_lower_amena = binom.test(n_viol_amena, tot_viol)$conf.int[1] * 100,

viol_CI_upper_amena = binom.test(n_viol_amena, tot_viol)$conf.int[2] * 100,

n_viol_fis = sum(PHYSICAL == "1"),

perc_viol_fis = n_viol_fis / tot_viol * 100,

viol_CI_lower_fis = binom.test(n_viol_fis, tot_viol)$conf.int[1] * 100,

viol_CI_upper_fis = binom.test(n_viol_fis, tot_viol)$conf.int[2] * 100,

n_viol_sex = sum(SEXUAL_VIOL == "1"),

perc_viol_sex = n_viol_sex / tot_viol * 100,

viol_CI_lower_sex = binom.test(n_viol_sex, tot_viol)$conf.int[1] * 100,

viol_CI_upper_sex = binom.test(n_viol_sex, tot_viol)$conf.int[2] * 100

)

exhaust <- all %>%

group_by(EXHAUST) %>%

summarise(

tot_viol = n(),

perc = n() / 4951 *100,

n_viol = sum(VIOLENCE == "1"),

perc_viol = n_viol / tot_viol * 100,

viol_CI_lower = binom.test(n_viol, tot_viol)$conf.int[1] * 100,

viol_CI_upper = binom.test(n_viol, tot_viol)$conf.int[2] * 100,

n_viol_insult = sum(INSULT == "1"),

perc_viol_insult = n_viol_insult / tot_viol * 100,

viol_CI_lower_insult = binom.test(n_viol_insult, tot_viol)$conf.int[1] * 100,

viol_CI_upper_insult = binom.test(n_viol_insult, tot_viol)$conf.int[2] * 100,

n_viol_amena = sum(AMENA == "1"),

perc_viol_amena = n_viol_amena / tot_viol * 100,

viol_CI_lower_amena = binom.test(n_viol_amena, tot_viol)$conf.int[1] * 100,

viol_CI_upper_amena = binom.test(n_viol_amena, tot_viol)$conf.int[2] * 100,

n_viol_fis = sum(PHYSICAL == "1"),

perc_viol_fis = n_viol_fis / tot_viol * 100,

viol_CI_lower_fis = binom.test(n_viol_fis, tot_viol)$conf.int[1] * 100,

viol_CI_upper_fis = binom.test(n_viol_fis, tot_viol)$conf.int[2] * 100,

n_viol_sex = sum(SEXUAL_VIOL == "1"),

perc_viol_sex = n_viol_sex / tot_viol * 100,

viol_CI_lower_sex = binom.test(n_viol_sex, tot_viol)$conf.int[1] * 100,

viol_CI_upper_sex = binom.test(n_viol_sex, tot_viol)$conf.int[2] * 100

)

deperso <- all %>%

group_by(DEPERSO) %>%

summarise(

tot_viol = n(),

perc = n() / 4951 *100,

n_viol = sum(VIOLENCE == "1"),

perc_viol = n_viol / tot_viol * 100,

viol_CI_lower = binom.test(n_viol, tot_viol)$conf.int[1] * 100,

viol_CI_upper = binom.test(n_viol, tot_viol)$conf.int[2] * 100,

n_viol_insult = sum(INSULT == "1"),

perc_viol_insult = n_viol_insult / tot_viol * 100,

viol_CI_lower_insult = binom.test(n_viol_insult, tot_viol)$conf.int[1] * 100,

viol_CI_upper_insult = binom.test(n_viol_insult, tot_viol)$conf.int[2] * 100,

n_viol_amena = sum(AMENA == "1"),

perc_viol_amena = n_viol_amena / tot_viol * 100,

viol_CI_lower_amena = binom.test(n_viol_amena, tot_viol)$conf.int[1] * 100,

viol_CI_upper_amena = binom.test(n_viol_amena, tot_viol)$conf.int[2] * 100,

n_viol_fis = sum(PHYSICAL == "1"),

perc_viol_fis = n_viol_fis / tot_viol * 100,

viol_CI_lower_fis = binom.test(n_viol_fis, tot_viol)$conf.int[1] * 100,

viol_CI_upper_fis = binom.test(n_viol_fis, tot_viol)$conf.int[2] * 100,

n_viol_sex = sum(SEXUAL_VIOL == "1"),

perc_viol_sex = n_viol_sex / tot_viol * 100,

viol_CI_lower_sex = binom.test(n_viol_sex, tot_viol)$conf.int[1] * 100,

viol_CI_upper_sex = binom.test(n_viol_sex, tot_viol)$conf.int[2] * 100

)

# Estimate prevalences

calculate_prevalence_ci <- function(count, total) {

prevalence <- count / total

error_margin <- 1.96 * sqrt((prevalence * (1 - prevalence)) / total)

lower_ci <- prevalence - error_margin

upper_ci <- prevalence + error_margin

return(c(prevalence, lower_ci, upper_ci))

}

# Assuming your data frame is named 'data'

prevalence_table <- all %>%

# group_by()

summarise(

Total = n(),

Count_VIOL = sum(VIOLENCE, na.rm = TRUE),

Count_DEP = sum(DEP, na.rm = TRUE),

Count_Work_Life_Balance = sum(WORK_LIFE_BALANCE_RECODED, na.rm = TRUE),

Count_SE = sum(SE, na.rm = TRUE),

Count_DEPERSO = sum(DEPERSO, na.rm = TRUE),

Count_EXHAUST = sum(EXHAUST, na.rm = TRUE),

Count_JENK = sum(JENK, na.rm = TRUE),

Count_QUIT = sum(LEAVE_INTENTION, na.rm = TRUE)

) %>%

rowwise() %>%

mutate(

Prevalence_VIOL = calculate_prevalence_ci(Count_VIOL, Total)[1] * 100,

CI_VIOL_Lower = calculate_prevalence_ci(Count_VIOL, Total)[2] * 100,

CI_viol_Upper = calculate_prevalence_ci(Count_VIOL, Total)[3] * 100,

Prevalence_DEP = calculate_prevalence_ci(Count_DEP, Total)[1] * 100,

CI_DEP_Lower = calculate_prevalence_ci(Count_DEP, Total)[2] * 100,

CI_DEP_Upper = calculate_prevalence_ci(Count_DEP, Total)[3] * 100,

Prevalence_Work_Life_Balance = calculate_prevalence_ci(Count_Work_Life_Balance, Total)[1] * 100,

CI_Work_Life_Balance_Lower = calculate_prevalence_ci(Count_Work_Life_Balance, Total)[2] * 100,

CI_Work_Life_Balance_Upper = calculate_prevalence_ci(Count_Work_Life_Balance, Total)[3] * 100,

Prevalence_SE = calculate_prevalence_ci(Count_SE, Total)[1] * 100,

CI_SE_Lower = calculate_prevalence_ci(Count_SE, Total)[2] * 100,

CI_SE_Upper = calculate_prevalence_ci(Count_SE, Total)[3] * 100,

Prevalence_DEPERSO = calculate_prevalence_ci(Count_DEPERSO, Total)[1] * 100,

CI_DEPERSO_Lower = calculate_prevalence_ci(Count_DEPERSO, Total)[2] * 100,

CI_DEPERSO_Upper = calculate_prevalence_ci(Count_DEPERSO, Total)[3] * 100,

Prevalence_EXHAUST = calculate_prevalence_ci(Count_EXHAUST, Total)[1] * 100,

CI_EXHAUST_Lower = calculate_prevalence_ci(Count_EXHAUST, Total)[2] * 100,

CI_EXHAUST_Upper = calculate_prevalence_ci(Count_EXHAUST, Total)[3] * 100,

Prevalence_JENK = calculate_prevalence_ci(Count_JENK, Total)[1] * 100,

CI_JENK_Lower = calculate_prevalence_ci(Count_JENK, Total)[2] * 100,

CI_JENK_Upper = calculate_prevalence_ci(Count_JENK, Total)[3] * 100,

Prevalence_QUIT = calculate_prevalence_ci(Count_QUIT, Total)[1] * 100,

CI_QUIT_Lower = calculate_prevalence_ci(Count_QUIT, Total)[2] * 100,

CI_QUIT_Upper = calculate_prevalence_ci(Count_QUIT, Total)[3] * 100

)

prevalence_table # Then adjust for each category

# Merge the set

work_life

se

insomnia

deper

depresion

exhaust

int_leave

## Create the prevalence plot

## VARIABLES FOR THE MODEL

# SEX

# MARITAL_STAT

# FAMILY_SUPPORT

# STABILITY

# YEAR OF PRACTICE

# VIOLENCE

# DEP | DEPRESSION

# HOURS-FULL

# SLEEP ADQ

# WORK LIFE BALANCE

# NEW DATASET

doctor <- all %>%

filter(JOB == "DOCTOR")

nurse <- all %>%

filter(JOB == "NURSE")

# Realiza los modelos no mtached PSM - usarás PSM para hacer todos los modelos

non_psm <- all

non_psm_d <- doctor

non_psm_n <- nurse

#########################

## NON-PSM FOR ALL ##

#########################

# DEPRESSION

non_psm %>%

group_by(DEP) %>%

summarise(

perc = n() / 4951 *100) # Prevalence of 26.8

model_phq2 <- glm(DEP ~ VIOLENCE + AGE + JOB + SEX + LEVEL_OF_CARE + MARITAL_STAT + FAMILY_SUPPORT + STABILITY +

YEARS_OF_PRACTICE + HOURS_WEEKLY +

HIGH_TRAIN + DEPARTMENT + PAID, data = non_psm,

family = poisson)

summary(model_phq2)

check_collinearity(model_phq2)

performance(model_phq2)

model_phq2_tab <- broom::tidy(model_phq2, conf.int = TRUE, exponentiate = TRUE)

print(model_phq2_tab, n=2)

# LEAVE_INTENTION DICHOTOMIC

non_psm %>%

group_by(LEAVE_INTENTION) %>%

summarise(

perc = n() / 4951 *100) # Prevalence of 28.2

model_leav <- glm(LEAVE_INTENTION ~ VIOLENCE + JOB + AGE + SEX + LEVEL_OF_CARE + MARITAL_STAT + FAMILY_SUPPORT + STABILITY +

YEARS_OF_PRACTICE + HOURS_WEEKLY +

HIGH_TRAIN + DEPARTMENT + PAID, data = non_psm,

family = poisson)

summary(model_leav)

check_collinearity(model_leav)

performance(model_leav)

model_leav_tab <- broom::tidy(model_leav, conf.int = TRUE, exponentiate = TRUE)

print(model_leav_tab, n=2)

# SELF_EFFICACY OUTCOME 1 = BELOW AVERAGE - poisson | BECAUSE LOW PREVALENCE

non_psm %>%

group_by(SE) %>%

summarise(

perc = n() / 4951 *100) # Prevalence of 2.67

model_self_ef <- glm(SE ~ VIOLENCE + JOB + AGE + SEX + LEVEL_OF_CARE + MARITAL_STAT + FAMILY_SUPPORT + STABILITY +

YEARS_OF_PRACTICE + HOURS_WEEKLY +

HIGH_TRAIN + DEPARTMENT + PAID, data = non_psm,

family = poisson)

summary(model_self_ef)

check_collinearity(model_self_ef)

performance(model_self_ef)

model_self_ef_tab <- broom::tidy(model_self_ef, conf.int = TRUE, exponentiate = TRUE)

print(model_self_ef_tab, n=2)

# DEPERSO 1 = BELOW AVERAGE

non_psm %>%

group_by(DEPERSO) %>%

summarise(

perc = n() / 4951 *100) # Prevalence of 37.1

glimpse(no_psm)

model_deperso <- glm(DEPERSO ~ VIOLENCE + JOB + AGE + SEX + LEVEL_OF_CARE + MARITAL_STAT + FAMILY_SUPPORT + STABILITY +

YEARS_OF_PRACTICE + HOURS_WEEKLY +

HIGH_TRAIN + DEPARTMENT + PAID, data = non_psm,

family = poisson)

summary(model_deperso)

check_collinearity(model_deperso)

performance(model_deperso)

model_deperso_tab <- broom::tidy(model_deperso, conf.int = TRUE, exponentiate = TRUE)

print(model_deperso_tab, n=2)

# EXHAUST 1 = BELOW AVERAGE

non_psm %>%

group_by(EXHAUST) %>%

summarise(

perc = n() / 4951 *100) # Prevalence of 26.8

model_exhaust <- glm(EXHAUST ~ VIOLENCE + JOB + AGE + SEX + LEVEL_OF_CARE + MARITAL_STAT + FAMILY_SUPPORT + STABILITY +

YEARS_OF_PRACTICE + HOURS_WEEKLY +

HIGH_TRAIN + DEPARTMENT + PAID, data = non_psm,

family = poisson)

summary(model_exhaust)

check_collinearity(model_exhaust)

performance(model_exhaust)

model_exhaust_tab <- broom::tidy(model_exhaust, conf.int = TRUE, exponentiate = TRUE)

print(model_exhaust_tab, n=2)

# SLEEP PROBLEM - JENKIN

non_psm %>%

group_by(JENK) %>%

summarise(

perc = n() / 4951 *100) # Prevalence of 5.59

model_insomn <- glm(JENK ~ VIOLENCE + AGE + JOB + SEX + LEVEL_OF_CARE + MARITAL_STAT + FAMILY_SUPPORT + STABILITY +

YEARS_OF_PRACTICE + HOURS_WEEKLY +

HIGH_TRAIN + DEPARTMENT + PAID, data = non_psm,

family = poisson)

summary(model_insomn)

check_collinearity(model_insomn)

performance(model_insomn)

model_insomn_tab <- broom::tidy(model_insomn, conf.int = TRUE, exponentiate = TRUE)

print(model_insomn_tab, n=2)

# WORK_LIFE_BALANCE = 1 | NO

non_psm %>%

group_by(WORK_LIFE_BALANCE_RECODED) %>%

summarise(

perc = n() / 4951 *100) # Prevalence of 39.1

model_work_life <- glm(WORK_LIFE_BALANCE_RECODED ~ VIOLENCE + JOB + AGE + SEX + LEVEL_OF_CARE + MARITAL_STAT + FAMILY_SUPPORT + STABILITY +

YEARS_OF_PRACTICE + HOURS_WEEKLY +

HIGH_TRAIN + DEPARTMENT + PAID, data = non_psm,

family = poisson)

summary(model_work_life)

check_collinearity(model_work_life)

performance(model_work_life)

model_work_life_tab <- broom::tidy(model_work_life, conf.int = TRUE, exponentiate = TRUE)

print(model_work_life_tab, n=2)

########################

## NON-PSM FOR NURSES ##

########################

# DEPRESSION

non_psm_n %>%

group_by(DEP) %>%

summarise(

perc = n() / 2826 *100) # 26.7 Prevalence

model_phq2_n <- glm(DEP ~ VIOLENCE + AGE + SEX + LEVEL_OF_CARE + MARITAL_STAT + FAMILY_SUPPORT + STABILITY +

YEARS_OF_PRACTICE + HOURS_WEEKLY +

HIGH_TRAIN + DEPARTMENT + PAID, data = non_psm_n,

family = poisson)

summary(model_phq2_n)

check_collinearity(model_phq2_n)

performance(model_phq2_n)

model_phq2_n_tab <- broom::tidy(model_phq2_n, conf.int = TRUE, exponentiate = TRUE)

print(model_phq2_n_tab, n=2)

# LEAVE_INTENTION DICHOTMOIC

non_psm_n %>%

group_by(LEAVE_INTENTION) %>%

summarise(

perc = n() / 2826 *100) # Prevalence of 13.8

model_leav_n <- glm(LEAVE_INTENTION ~ VIOLENCE + AGE + SEX + LEVEL_OF_CARE + MARITAL_STAT + FAMILY_SUPPORT + STABILITY +

YEARS_OF_PRACTICE + HOURS_WEEKLY +

HIGH_TRAIN + DEPARTMENT + PAID, data = non_psm_n,

family = poisson)

summary(model_leav_n)

check_collinearity(model_leav_n)

performance(model_leav_n)

model_leav_n_tab <- broom::tidy(model_leav_n, conf.int = TRUE, exponentiate = TRUE)

print(model_leav_n_tab, n=2)

# SELF_EFFICACY OUTCOME 1 = BELOW AVERAGE - poisson | BECAUSE LOW PREVALENCE

non_psm_n %>%

group_by(SE) %>%

summarise(

perc = n() / 2826 *100) # Prevalence of 2.44

model_self_ef_n <- glm(SE ~ VIOLENCE + AGE + SEX + LEVEL_OF_CARE + MARITAL_STAT + FAMILY_SUPPORT + STABILITY +

YEARS_OF_PRACTICE + HOURS_WEEKLY +

HIGH_TRAIN + DEPARTMENT + PAID, data = non_psm_n,

family = poisson)

summary(model_self_ef_n)

check_collinearity(model_self_ef_n)

performance(model_self_ef_n)

exp(0.72764)

2.070189 - 1.96 * 0.24352

2.070189 + 1.96 * 0.24352

model_self_ef_n_tab <- broom::tidy(model_self_ef_n, conf.int = TRUE, exponentiate = TRUE)

model_self_ef_n_tab

# DEPERSO 1 = BELOW AVERAGE

non_psm_n %>%

group_by(DEPERSO) %>%

summarise(

perc = n() / 2826 *100) # Prevalence of 37.1

model_deperso_n <- glm(DEPERSO ~ VIOLENCE + AGE + SEX + LEVEL_OF_CARE + MARITAL_STAT + FAMILY_SUPPORT + STABILITY +

YEARS_OF_PRACTICE + HOURS_WEEKLY +

HIGH_TRAIN + DEPARTMENT + PAID, data = non_psm_n,

family = poisson)

summary(model_deperso_n)

check_collinearity(model_deperso_n)

performance(model_deperso_n)

model_deperso_n_tab <- broom::tidy(model_deperso_n, conf.int = TRUE, exponentiate = TRUE)

model_deperso_n_tab

# EXHAUST 1 = BELOW AVERAGE

non_psm_n %>%

group_by(EXHAUST) %>%

summarise(

perc = n() / 2826 *100) # Prevalence of 21.4 in nurses

model_exhaust_n <- glm(EXHAUST ~ VIOLENCE + AGE + SEX + LEVEL_OF_CARE + MARITAL_STAT + FAMILY_SUPPORT + STABILITY +

YEARS_OF_PRACTICE + HOURS_WEEKLY +

HIGH_TRAIN + DEPARTMENT + PAID, data = non_psm_n,

family = poisson)

summary(model_exhaust_n)

check_collinearity(model_exhaust_n)

performance(model_exhaust_n)

model_exhaust_n_tab <- broom::tidy(model_exhaust_n, conf.int = TRUE, exponentiate = TRUE)

model_exhaust_n_tab

# SLEEP PROBLEM - JENKIN

non_psm_n %>%

group_by(JENK) %>%

summarise(

perc = n() / 2826 *100) # Prevalence of 4.85

model_insomn_n <- glm(JENK ~ VIOLENCE + AGE + SEX + LEVEL_OF_CARE + MARITAL_STAT + FAMILY_SUPPORT + STABILITY +

YEARS_OF_PRACTICE + HOURS_WEEKLY +

HIGH_TRAIN + DEPARTMENT + PAID, data = non_psm_n,

family = poisson)

summary(model_insomn_n)

check_collinearity(model_insomn_n)

performance(model_insomn_n)

model_insomn_n_tab <- broom::tidy(model_insomn_n, conf.int = TRUE, exponentiate = TRUE)

model_insomn_n_tab

# WORK_LIFE_BALANCE = 1 | NO

non_psm_n %>%

group_by(WORK_LIFE_BALANCE_RECODED) %>%

summarise(

perc = n() / 2826 *100) # Prevalence of 33.0

model_work_life_n <- glm(WORK_LIFE_BALANCE_RECODED ~ VIOLENCE + AGE + SEX + LEVEL_OF_CARE + MARITAL_STAT + FAMILY_SUPPORT + STABILITY +

YEARS_OF_PRACTICE + HOURS_WEEKLY +

HIGH_TRAIN + DEPARTMENT + PAID, data = non_psm_n,

family = poisson)

summary(model_work_life_n)

check_collinearity(model_work_life_n)

performance(model_work_life_n)

model_work_life_n_tab <- broom::tidy(model_work_life_n, conf.int = TRUE, exponentiate = TRUE)

model_work_life_n_tab

#########################

## NON-PSM FOR DOCTORS ##

#########################

# DEPRESSION

non_psm_d %>%

group_by(DEP) %>%

summarise(

perc = n() / 2125 *100) # 27.0 Prevalence

model_phq2_d <- glm(DEP ~ VIOLENCE + AGE + SEX + LEVEL_OF_CARE + MARITAL_STAT + FAMILY_SUPPORT + STABILITY +

YEARS_OF_PRACTICE + HOURS_WEEKLY +

HIGH_TRAIN + DEPARTMENT + PAID, data = non_psm_d,

family = poisson)

summary(model_phq2_d)

check_collinearity(model_phq2_d)

performance(model_phq2_d)

model_phq2_d_tab <- broom::tidy(model_phq2_d, conf.int = TRUE, exponentiate = TRUE)

print(model_phq2_d_tab, n=2)

# LEAVE_INTENTION DICHOTMOIC

non_psm_d %>%

group_by(LEAVE_INTENTION) %>%

summarise(

perc = n() / 2125 *100) # Prevalence of 13.8

model_leav_d <- glm(LEAVE_INTENTION ~ VIOLENCE + AGE + SEX + LEVEL_OF_CARE + MARITAL_STAT + FAMILY_SUPPORT + STABILITY +

YEARS_OF_PRACTICE + HOURS_WEEKLY +

HIGH_TRAIN + DEPARTMENT + PAID, data = non_psm_d,

family = poisson)

summary(model_leav_d)

check_collinearity(model_leav_d)

performance(model_leav_d)

model_leav_d_tab <- broom::tidy(model_leav_d, conf.int = TRUE, exponentiate = TRUE)

print(model_leav_d_tab, n=2)

# SELF_EFFICACY OUTCOME 1 = BELOW AVERAGE - poisson | BECAUSE LOW PREVALENCE

non_psm_d %>%

group_by(SE) %>%

summarise(

perc = n() / 2125 *100) # Prevalence of 2.96

model_self_ef_d <- glm(SE ~ VIOLENCE + AGE + SEX + LEVEL_OF_CARE + MARITAL_STAT + FAMILY_SUPPORT + STABILITY +

YEARS_OF_PRACTICE + HOURS_WEEKLY +

HIGH_TRAIN + DEPARTMENT + PAID, data = non_psm_d,

family = poisson)

summary(model_self_ef_d)

check_collinearity(model_self_ef_d)

performance(model_self_ef_d)

model_self_ef_d_tab <- broom::tidy(model_self_ef_d, conf.int = TRUE, exponentiate = TRUE)

model_self_ef_d_tab

# DEPERSO 1 = BELOW AVERAGE

non_psm_d %>%

group_by(DEPERSO) %>%

summarise(

perc = n() / 2125 *100) # Prevalence of 44.9

model_deperso_d <- glm(DEPERSO ~ VIOLENCE + AGE + SEX + LEVEL_OF_CARE + MARITAL_STAT + FAMILY_SUPPORT + STABILITY +

YEARS_OF_PRACTICE + HOURS_WEEKLY +

HIGH_TRAIN + DEPARTMENT + PAID, data = non_psm_d,

family = poisson)

summary(model_deperso_d)

check_collinearity(model_deperso_d)

performance(model_deperso_d)

model_deperso_d_tab <- broom::tidy(model_deperso_d, conf.int = TRUE, exponentiate = TRUE)

model_deperso_d_tab

# EXHAUST 1 = BELOW AVERAGE

non_psm_d %>%

group_by(EXHAUST) %>%

summarise(

perc = n() / 2125 *100) # Prevalence of 21.4 in nurses

model_exhaust_d <- glm(EXHAUST ~ VIOLENCE + AGE + SEX + LEVEL_OF_CARE + MARITAL_STAT + FAMILY_SUPPORT + STABILITY +

YEARS_OF_PRACTICE + HOURS_WEEKLY +

HIGH_TRAIN + DEPARTMENT + PAID, data = non_psm_d,

family = poisson)

summary(model_exhaust_d)

check_collinearity(model_exhaust_d)

performance(model_exhaust_d)

model_exhaust_d_tab <- broom::tidy(model_exhaust_d, conf.int = TRUE, exponentiate = TRUE)

model_exhaust_d_tab

# SLEEP PROBLEM - JENKIN

non_psm_d %>%

group_by(JENK) %>%

summarise(

perc = n() / 2125 *100) # Prevalence of 6.59

model_insomn_d <- glm(JENK ~ VIOLENCE + AGE + SEX + LEVEL_OF_CARE + MARITAL_STAT + FAMILY_SUPPORT + STABILITY +

YEARS_OF_PRACTICE + HOURS_WEEKLY +

HIGH_TRAIN + DEPARTMENT + PAID, data = non_psm_d,

family = poisson)

summary(model_insomn_d)

check_collinearity(model_insomn_d)

performance(model_insomn_d)

model_insomn_d_tab <- broom::tidy(model_insomn_d, conf.int = TRUE, exponentiate = TRUE)

model_insomn_d_tab

# WORK_LIFE_BALANCE = 1 | NO

non_psm_d %>%

group_by(WORK_LIFE_BALANCE_RECODED) %>%

summarise(

perc = n() / 2125 *100) # Prevalence of 47.1

model_work_life_d <- glm(WORK_LIFE_BALANCE_RECODED ~ VIOLENCE + AGE + SEX + LEVEL_OF_CARE + MARITAL_STAT + FAMILY_SUPPORT + STABILITY +

YEARS_OF_PRACTICE + HOURS_WEEKLY +

HIGH_TRAIN + DEPARTMENT + PAID, data = non_psm_d,

family = poisson)

summary(model_work_life_d)

check_collinearity(model_work_life_d)

performance(model_work_life_d)

model_work_life_d_tab <- broom::tidy(model_work_life_d, conf.int = TRUE, exponentiate = TRUE)

model_work_life_d_tab

#########################

### DO THE PSM MODELS ###

#########################

####### PMS FOR ALL #########

# Aquí, 'TREATMENT' debe ser la variable binaria de tratamiento (0 o 1)

psm_all <- matchit( VIOLENCE ~ AGE + JOB + SEX + LEVEL_OF_CARE + MARITAL_STAT + FAMILY_SUPPORT + STABILITY +

YEARS_OF_PRACTICE + HOURS_WEEKLY +

HIGH_TRAIN + DEPARTMENT + PAID,

data = non_psm, method = "nearest", ratio = 1)

matched_data <- match.data(psm_all)

# Check the model for balance

balance <- bal.tab(psm_all)

print(balance)

love.plot(balance, threshold = 0.1)

# Create the table to compare

vars <- c("AGE", "SEX", "JOB", "HIGH_TRAIN", "LEVEL_OF_CARE",

"MARITAL_STAT", "FAMILY_SUPPORT", "STABILITY",

"YEARS_OF_PRACTICE", "HOURS_WEEKLY", "PAID", "DEPARTMENT")

table1_non_psm <- CreateTableOne(vars = vars, strata = "VIOLENCE", data = non_psm, test = TRUE)

table1_non_psm

table1_psm <- CreateTableOne(vars = vars, strata = "VIOLENCE", data = matched_data, test = TRUE)

table1_psm

##########

########## PSM FOR DOCTORS ##########

psm_doctor <- matchit( VIOLENCE ~ AGE + SEX + LEVEL_OF_CARE + MARITAL_STAT + FAMILY_SUPPORT + STABILITY +

YEARS_OF_PRACTICE + HOURS_WEEKLY +

HIGH_TRAIN + DEPARTMENT + PAID,

data = non_psm_d, method = "nearest", ratio = 1)

matched_data_doc <- match.data(psm_doctor)

# Check the model for balance

balance_doc <- bal.tab(psm_doctor)

print(balance_doc)

love.plot(balance_doc, threshold = 0.1)

# Create the table to compare

vars <- c("AGE", "SEX", "JOB", "HIGH_TRAIN", "LEVEL_OF_CARE",

"MARITAL_STAT", "FAMILY_SUPPORT", "STABILITY",

"YEARS_OF_PRACTICE", "HOURS_WEEKLY", "PAID", "DEPARTMENT")

table1_non_psm_doc <- CreateTableOne(vars = vars, strata = "VIOLENCE", data = non_psm_d, test = TRUE)

table1_non_psm_doc

table1_psm_doc <- CreateTableOne(vars = vars, strata = "VIOLENCE", data = matched_data_doc, test = TRUE)

table1_psm_doc

########

########## PSM FOR NURSES ##########

psm_nurse <- matchit( VIOLENCE ~ AGE + SEX + LEVEL_OF_CARE + MARITAL_STAT + FAMILY_SUPPORT + STABILITY +

YEARS_OF_PRACTICE + HOURS_WEEKLY +

HIGH_TRAIN + DEPARTMENT + PAID,

data = non_psm_n, method = "nearest", ratio = 1)

matched_data_nurse <- match.data(psm_nurse)

# Check the model for balance

balance <- bal.tab(psm_nurse)

print(balance)

love.plot(balance, threshold = 0.1)

# Create the table to compare

vars <- c("AGE", "SEX", "JOB", "HIGH_TRAIN", "LEVEL_OF_CARE",

"MARITAL_STAT", "FAMILY_SUPPORT", "STABILITY",

"YEARS_OF_PRACTICE", "HOURS_WEEKLY", "PAID", "DEPARTMENT")

table1_non_psm_nur <- CreateTableOne(vars = vars, strata = "VIOLENCE", data = non_psm_n, test = TRUE)

table1_non_psm_nur

table1_psm_nur <- CreateTableOne(vars = vars, strata = "VIOLENCE", data = matched_data_nurse, test = TRUE)

table1_psm_nur

##################################################

##### PSM MODELS FOR ALL - DOCTOR AN NURSES ######

##################################################

# You will use: matched_data, matched_data_doc, matched_data_nurse

############# MATCHED DATA ###################

# DEPRESSION

matched_data %>%

group_by(DEP) %>%

summarise(

perc = n() / 4150 *100) # Prevalence of 23.5

model_phq2 <- glm(DEP ~ VIOLENCE + AGE + JOB + SEX + LEVEL_OF_CARE + MARITAL_STAT + FAMILY_SUPPORT + STABILITY +

YEARS_OF_PRACTICE + HOURS_WEEKLY +

HIGH_TRAIN + DEPARTMENT + PAID, data = matched_data,

family = poisson)

summary(model_phq2)

check_collinearity(model_phq2)

performance(model_phq2)

model_phq2_tab <- broom::tidy(model_phq2, conf.int = TRUE, exponentiate = TRUE)

print(model_phq2_tab, n=2)

# LEAVE_INTENTION DICHOTMOIC

matched_data %>%

group_by(LEAVE_INTENTION) %>%

summarise(

perc = n() / 4150 *100) # Prevalence of 24.6

model_leav <- glm(LEAVE_INTENTION ~ VIOLENCE + JOB + AGE + SEX + LEVEL_OF_CARE + MARITAL_STAT + FAMILY_SUPPORT + STABILITY +

YEARS_OF_PRACTICE + HOURS_WEEKLY +

HIGH_TRAIN + DEPARTMENT + PAID, data = matched_data,

family = poisson)

summary(model_leav)

check_collinearity(model_leav)

performance(model_leav)

model_leav_tab <- broom::tidy(model_leav, conf.int = TRUE, exponentiate = TRUE)

print(model_leav_tab, n=2)

# SELF_EFFICACY OUTCOME 1 = BELOW AVERAGE - poisson | BECAUSE LOW PREVALENCE

matched_data %>%

group_by(SE) %>%

summarise(

perc = n() / 4150 *100) # Prevalence of 2.32

model_self_ef <- glm(SE ~ VIOLENCE + JOB + AGE + SEX + LEVEL_OF_CARE + MARITAL_STAT + FAMILY_SUPPORT + STABILITY +

YEARS_OF_PRACTICE + HOURS_WEEKLY +

HIGH_TRAIN + DEPARTMENT + PAID, data = matched_data,

family = poisson)

summary(model_self_ef)

check_collinearity(model_self_ef)

performance(model_self_ef)

model_self_ef_tab <- broom::tidy(model_self_ef, conf.int = TRUE, exponentiate = TRUE)

model_self_ef_tab

# DEPERSO 1 = BELOW AVERAGE

matched_data %>%

group_by(DEPERSO) %>%

summarise(

perc = n() / 4150 *100) # Prevalence of 32.7

glimpse(no_psm)

model_deperso <- glm(DEPERSO ~ VIOLENCE + JOB + AGE + SEX + LEVEL_OF_CARE + MARITAL_STAT + FAMILY_SUPPORT + STABILITY +

YEARS_OF_PRACTICE + HOURS_WEEKLY +

HIGH_TRAIN + DEPARTMENT + PAID, data = matched_data,

family = poisson)

summary(model_deperso)

check_collinearity(model_deperso)

performance(model_deperso)

model_deperso_tab <- broom::tidy(model_deperso, conf.int = TRUE, exponentiate = TRUE)

model_deperso_tab

# EXHAUST 1 = BELOW AVERAGE

matched_data %>%

group_by(EXHAUST) %>%

summarise(

perc = n() / 4150 *100) # Prevalence of 23.9

model_exhaust <- glm(EXHAUST ~ VIOLENCE + JOB + AGE + SEX + LEVEL_OF_CARE + MARITAL_STAT + FAMILY_SUPPORT + STABILITY +

YEARS_OF_PRACTICE + HOURS_WEEKLY +

HIGH_TRAIN + DEPARTMENT + PAID, data = matched_data,

family = poisson)

summary(model_exhaust)

check_collinearity(model_exhaust)

performance(model_exhaust)

model_exhaust_tab <- broom::tidy(model_exhaust, conf.int = TRUE, exponentiate = TRUE)

model_exhaust_tab

# SLEEP PROBLEM - JENKIN

matched_data %>%

group_by(JENK) %>%

summarise(

perc = n() / 4150 *100) # Prevalence of 5.59

model_insomn <- glm(JENK ~ VIOLENCE + AGE + JOB + SEX + LEVEL_OF_CARE + MARITAL_STAT + FAMILY_SUPPORT + STABILITY +

YEARS_OF_PRACTICE + HOURS_WEEKLY +

HIGH_TRAIN + DEPARTMENT + PAID, data = matched_data,

family = poisson)

summary(model_insomn)

check_collinearity(model_insomn)

performance(model_insomn)

model_insomn_tab <- broom::tidy(model_insomn, conf.int = TRUE, exponentiate = TRUE)

model_insomn_tab

# WORK_LIFE_BALANCE = 1 | NO

matched_data %>%

group_by(WORK_LIFE_BALANCE_RECODED) %>%

summarise(

perc = n() / 4150 *100) # Prevalence of 39.1

model_work_life <- glm(WORK_LIFE_BALANCE_RECODED ~ VIOLENCE + JOB + AGE + SEX + LEVEL_OF_CARE + MARITAL_STAT + FAMILY_SUPPORT + STABILITY +

YEARS_OF_PRACTICE + HOURS_WEEKLY +

HIGH_TRAIN + DEPARTMENT + PAID, data = matched_data,

family = poisson)

summary(model_work_life)

check_collinearity(model_work_life)

performance(model_work_life)

model_work_life_tab <- broom::tidy(model_work_life, conf.int = TRUE, exponentiate = TRUE)

model_work_life_tab

############# MATCHED DATA FOR NURSES ###################

# DEPRESSION

matched_data_nurse %>%

group_by(DEP) %>%

summarise(

perc = n() / 2134 *100) # Prevalence of 29.2

model_phq2 <- glm(DEP ~ VIOLENCE + AGE + SEX + LEVEL_OF_CARE + MARITAL_STAT + FAMILY_SUPPORT + STABILITY +

YEARS_OF_PRACTICE + HOURS_WEEKLY +

HIGH_TRAIN + DEPARTMENT + PAID, data = matched_data_nurse,

family = poisson)

summary(model_phq2)

check_collinearity(model_phq2)

performance(model_phq2)

model_phq2_tab <- broom::tidy(model_phq2, conf.int = TRUE, exponentiate = TRUE)

print(model_phq2_tab, n=2)

# LEAVE_INTENTION DICHOTOMIC - Poisson

matched_data_nurse %>%

group_by(LEAVE_INTENTION) %>%

summarise(

perc = n() / 2134 *100) # Prevalence of 24.1

model_leav <- glm(LEAVE_INTENTION ~ VIOLENCE + AGE + SEX + LEVEL_OF_CARE + MARITAL_STAT + FAMILY_SUPPORT + STABILITY +

YEARS_OF_PRACTICE + HOURS_WEEKLY +

HIGH_TRAIN + DEPARTMENT + PAID, data = matched_data_nurse,

family = poisson)

summary(model_leav)

check_collinearity(model_leav)

performance(model_leav)

model_leav_tab <- broom::tidy(model_leav, conf.int = TRUE, exponentiate = TRUE)

print(model_leav_tab, n=2)

# SELF_EFFICACY OUTCOME 1 = BELOW AVERAGE - poisson | BECAUSE LOW PREVALENCE

matched_data_nurse %>%

group_by(SE) %>%

summarise(

perc = n() / 2134 *100) # Prevalence of 2.58

model_self_ef_nur <- glm(SE ~ VIOLENCE + AGE + SEX + LEVEL_OF_CARE + MARITAL_STAT + FAMILY_SUPPORT + STABILITY +

YEARS_OF_PRACTICE + HOURS_WEEKLY +

HIGH_TRAIN + DEPARTMENT + PAID, data = matched_data_nurse,

family = poisson)

summary(model_self_ef_nur)

exp(0.65276)

1.920835 + 1.96 * 0.27701

1.920835 - 1.96 * 0.27701

check_collinearity(model_self_ef_nur)

performance(model_self_ef_nur)

model_self_ef_tab <- broom::tidy(model_self_ef_nur, conf.int = TRUE, exponentiate = TRUE)

model_self_ef_tab

# DEPERSO 1 = BELOW AVERAGE

matched_data_nurse %>%

group_by(DEPERSO) %>%

summarise(

perc = n() / 2134 *100) # Prevalence of 32.4

model_deperso <- glm(DEPERSO ~ VIOLENCE + AGE + SEX + LEVEL_OF_CARE + MARITAL_STAT + FAMILY_SUPPORT + STABILITY +

YEARS_OF_PRACTICE + HOURS_WEEKLY +

HIGH_TRAIN + DEPARTMENT + PAID, data = matched_data_nurse,

family = poisson)

summary(model_deperso)

check_collinearity(model_deperso)

performance(model_deperso)

model_deperso_tab <- broom::tidy(model_deperso, conf.int = TRUE, exponentiate = TRUE)

model_deperso_tab

# EXHAUST 1 = BELOW AVERAGE

matched_data_nurse %>%

group_by(EXHAUST) %>%

summarise(

perc = n() / 2134 *100) # Prevalence of 23.1

model_exhaust <- glm(EXHAUST ~ VIOLENCE + AGE + SEX + LEVEL_OF_CARE + MARITAL_STAT + FAMILY_SUPPORT + STABILITY +

YEARS_OF_PRACTICE + HOURS_WEEKLY +

HIGH_TRAIN + DEPARTMENT + PAID, data = matched_data_nurse,

family = poisson)

summary(model_exhaust)

check_collinearity(model_exhaust)

performance(model_exhaust)

model_exhaust_tab <- broom::tidy(model_exhaust, conf.int = TRUE, exponentiate = TRUE)

model_exhaust_tab

# SLEEP PROBLEM - JENKIN

matched_data_nurse %>%

group_by(JENK) %>%

summarise(

perc = n() / 2134 *100) # Prevalence of 5.39

model_insomn <- glm(JENK ~ VIOLENCE + AGE + SEX + LEVEL_OF_CARE + MARITAL_STAT + FAMILY_SUPPORT + STABILITY +

YEARS_OF_PRACTICE + HOURS_WEEKLY +

HIGH_TRAIN + DEPARTMENT + PAID, data = matched_data_nurse,

family = poisson)

summary(model_insomn)

check_collinearity(model_insomn)

performance(model_insomn)

model_insomn_tab <- broom::tidy(model_insomn, conf.int = TRUE, exponentiate = TRUE)

model_insomn_tab

# WORK_LIFE_BALANCE = 1 | NO

matched_data_nurse %>%

group_by(WORK_LIFE_BALANCE_RECODED) %>%

summarise(

perc = n() / 2134 *100) # Prevalence of 34.2

model_work_life <- glm(WORK_LIFE_BALANCE_RECODED ~ VIOLENCE + AGE + SEX + LEVEL_OF_CARE + MARITAL_STAT + FAMILY_SUPPORT + STABILITY +

YEARS_OF_PRACTICE + HOURS_WEEKLY +

HIGH_TRAIN + DEPARTMENT + PAID, data = matched_data_nurse,

family = poisson)

summary(model_work_life)

check_collinearity(model_work_life)

performance(model_work_life)

model_work_life_tab <- broom::tidy(model_work_life, conf.int = TRUE, exponentiate = TRUE)

model_work_life_tab

############ MATCHED DATA FOR DOCTORS ##############

# DEPRESSION

matched_data_doc %>%

group_by(DEP) %>%

summarise(

perc = n() / 2016 *100) # Prevalence of 23.5

model_phq2 <- glm(DEP ~ VIOLENCE + AGE + SEX + LEVEL_OF_CARE + MARITAL_STAT + FAMILY_SUPPORT + STABILITY +

YEARS_OF_PRACTICE + HOURS_WEEKLY +

HIGH_TRAIN + DEPARTMENT + PAID, data = matched_data_doc,

family = poisson)

summary(model_phq2)

check_collinearity(model_phq2)

performance(model_phq2)

model_phq2_tab <- broom::tidy(model_phq2, conf.int = TRUE, exponentiate = TRUE)

print(model_phq2_tab, n=2)

# LEAVE_INTENTION DICHOTOMIC - THIS WAS poisson DUE TO FAIL OF POISSON

matched_data_doc %>%

group_by(LEAVE_INTENTION) %>%

summarise(

perc = n() / 2016 *100) # Prevalence of 32.8

model_leav <- glm(LEAVE_INTENTION ~ VIOLENCE + AGE + SEX + LEVEL_OF_CARE + MARITAL_STAT + FAMILY_SUPPORT + STABILITY +

YEARS_OF_PRACTICE + HOURS_WEEKLY +

HIGH_TRAIN + DEPARTMENT + PAID, data = matched_data_doc,

family = poisson)

summary(model_leav)

check_collinearity(model_leav)

performance(model_leav)

model_leav_tab <- broom::tidy(model_leav, conf.int = TRUE, exponentiate = TRUE)

print(model_leav_tab, n=2)

# SELF_EFFICACY OUTCOME 1 = BELOW AVERAGE - poisson | BECAUSE LOW PREVALENCE

matched_data_doc %>%

group_by(SE) %>%

summarise(

perc = n() / 2016 *100) # Prevalence of 2.58

model_self_ef <- glm(SE ~ VIOLENCE + AGE + SEX + LEVEL_OF_CARE + MARITAL_STAT + FAMILY_SUPPORT + STABILITY +

YEARS_OF_PRACTICE + HOURS_WEEKLY +

HIGH_TRAIN + DEPARTMENT + PAID, data = matched_data_doc,

family = poisson)

summary(model_self_ef)

check_collinearity(model_self_ef)

performance(model_self_ef)

model_self_ef_tab <- broom::tidy(model_self_ef, conf.int = TRUE, exponentiate = TRUE)

model_self_ef_tab

# DEPERSO 1 = BELOW AVERAGE

matched_data_doc %>%

group_by(DEPERSO) %>%

summarise(

perc = n() / 2016 *100) # Prevalence of 32.4

model_deperso <- glm(DEPERSO ~ VIOLENCE + AGE + SEX + LEVEL_OF_CARE + MARITAL_STAT + FAMILY_SUPPORT + STABILITY +

YEARS_OF_PRACTICE + HOURS_WEEKLY +

HIGH_TRAIN + DEPARTMENT + PAID, data = matched_data_doc,

family = poisson)

summary(model_deperso)

check_collinearity(model_deperso)

performance(model_deperso)

model_deperso_tab <- broom::tidy(model_deperso, conf.int = TRUE, exponentiate = TRUE)

model_deperso_tab

# EXHAUST 1 = BELOW AVERAGE

matched_data_doc %>%

group_by(EXHAUST) %>%

summarise(

perc = n() / 2016 *100) # Prevalence of 35

model_exhaust <- glm(EXHAUST ~ VIOLENCE + AGE + SEX + LEVEL_OF_CARE + MARITAL_STAT + FAMILY_SUPPORT + STABILITY +

YEARS_OF_PRACTICE + HOURS_WEEKLY +

HIGH_TRAIN + DEPARTMENT + PAID, data = matched_data_doc,

family = poisson)

summary(model_exhaust)

check_collinearity(model_exhaust)

performance(model_exhaust)

model_exhaust_tab <- broom::tidy(model_exhaust, conf.int = TRUE, exponentiate = TRUE)

model_exhaust_tab

# SLEEP PROBLEM - JENKIN

matched_data_doc %>%

group_by(JENK) %>%

summarise(

perc = n() / 2016 *100) # Prevalence of 5.39

model_insomn <- glm(JENK ~ VIOLENCE + AGE + SEX + LEVEL_OF_CARE + MARITAL_STAT + FAMILY_SUPPORT + STABILITY +

YEARS_OF_PRACTICE + HOURS_WEEKLY +

HIGH_TRAIN + DEPARTMENT + PAID, data = matched_data_doc,

family = poisson)

summary(model_insomn)

check_collinearity(model_insomn)

performance(model_insomn)

model_insomn_tab <- broom::tidy(model_insomn, conf.int = TRUE, exponentiate = TRUE)

model_insomn_tab

# WORK_LIFE_BALANCE = 1 | NO

matched_data_doc %>%

group_by(WORK_LIFE_BALANCE_RECODED) %>%

summarise(

perc = n() / 2016 *100) # Prevalence of 34.2

model_work_life <- glm(WORK_LIFE_BALANCE_RECODED ~ VIOLENCE + AGE + SEX + LEVEL_OF_CARE + MARITAL_STAT + FAMILY_SUPPORT + STABILITY +

YEARS_OF_PRACTICE + HOURS_WEEKLY +

HIGH_TRAIN + DEPARTMENT + PAID, data = matched_data_doc,

family = poisson)

summary(model_work_life)

check_collinearity(model_work_life)

performance(model_work_life)

model_work_life_tab <- broom::tidy(model_work_life, conf.int = TRUE, exponentiate = TRUE)

model_work_life_tab

####################################

######## CREATE THE GRAPH ##########

####################################

###############################

### PREVALENCE RATIOS GRAPH ###

###############################

# Define the data

Parameter <- c(

"Depressive symptoms", "Intention to quit", "Low professional fulfillment",

"Depersonalization", "Emotional exhaustion", "Sleep problems", "No worklife balance",

"Depressive symptoms", "Intention to quit", "Low professional fulfillment",

"Depersonalization", "Emotional exhaustion", "Sleep problems", "No worklife balance",

"Depressive symptoms", "Intention to quit", "Low professional fulfillment",

"Depersonalization", "Emotional exhaustion", "Sleep problems", "No worklife balance",

"Depressive symptoms", "Intention to quit", "Low professional fulfillment",

"Depersonalization", "Emotional exhaustion", "Sleep problems", "No worklife balance",

"Depressive symptoms", "Intention to quit", "Low professional fulfillment",

"Depersonalization", "Emotional exhaustion", "Sleep problems", "No worklife balance",

"Depressive symptoms", "Intention to quit", "Low professional fulfillment",

"Depersonalization", "Emotional exhaustion", "Sleep problems", "No worklife balance"

)

Group <- c(

rep("Non PSM", 7), rep("Non PSM-nurse", 7), rep("Non PSM-doctor", 7),

rep("PSM", 7), rep("PSM-nurse", 7), rep("PSM-doctor", 7)

)

RR_CI <- c(

"1.64 (1.47 to 1.83)", "1.27 (1.14 to 1.41)", "1.45 (1.02 to 2.04)",

"1.57 (1.43 to 1.72)", "1.58 (1.42 to 1.77)", "2.18 (1.71 to 2.79)", "1.16 (1.07 to 1.27)",

"1.62 (1.41 to 1.87)", "1.20 (1.03 to 1.40)", "2.07 (1.59 to 2.54)",

"1.61 (1.41 to 1.83)", "1.48 (1.26 to 1.74)", "2.23 (1.59 to 3.14)", "1.16 (1.02 to 1.33)",

"1.67 (1.41 to 1.97)", "1.33 (1.14 to 1.54)", "1.04 (0.63 to 1.71)",

"1.53 (1.35 to 1.75)", "1.70 (1.46 to 1.97)", "2.14 (1.51 to 3.07)", "1.17 (1.04 to 1.33)",

"1.65 (1.47 to 1.86)", "1.26 (1.13 to 1.41)", "1.45 (1.00 to 2.12)",

"1.58 (1.43 to 1.75)", "1.59 (1.41 to 1.79)", "2.15 (1.65 to 2.81)", "1.18 (1.07 to 1.30)",

"1.62 (1.37 to 1.91)", "1.20 (1.01 to 1.43)", "1.92 (1.38 to 2.47)",

"1.67 (1.43 to 1.94)", "1.41 (1.18 to 1.70)", "2.18 (1.48 to 3.28)", "1.16 (1.00 to 1.34)",

"1.64 (1.39 to 1.95)", "1.33 (1.14 to 1.55)", "1.00 (0.64 to 1.66)",

"1.52 (1.33 to 1.74)", "1.69 (1.45 to 1.97)", "2.06 (1.45 to 2.97)", "1.18 (1.04 to 1.34)"

)

# Create the dataframe

dat <- data.frame(Parameter, Group, RR_CI)

# Separate the RR and the CI into different columns

dat <- dat %>%

separate(RR_CI, into = c("RR", "CI"), sep = " \\(", remove = FALSE) %>%

mutate(

CI = sub("\\)", "", CI),

Lower = as.numeric(sub(" to .*", "", CI)),

Upper = as.numeric(sub(".* to ", "", CI)),

RR = as.numeric(RR)

) %>%

select(-CI, -RR_CI)

# Convert the 'Parameter' column to a factor

dat$Parameter <- factor(dat$Parameter, levels=c(

"Depressive symptoms", "Intention to quit", "Low professional fulfillment",

"Depersonalization", "Emotional exhaustion", "Sleep problems",

"No worklife balance"

))

# Order the Group factor

dat$Group <- factor(dat$Group, levels=c("PSM", "Non PSM", "PSM-nurse", "Non PSM-nurse", "PSM-doctor", "Non PSM-doctor"))

levels_order <- c("PSM", "Non PSM", "PSM-nurse", "Non PSM-nurse", "PSM-doctor", "Non PSM-doctor")

reversed_levels_order <- rev(levels_order)

dat$Group <- factor(dat$Group, levels = reversed_levels_order)

# Plot

jpeg("Figure 2_Viol.jpg", width = 8.27, height = 11.69, units = "in", res = 300)

ggplot(data=dat, aes(x=Group, y=RR, ymin=Lower, ymax=Upper, color=Group)) +

geom_pointrange(aes(shape=Group), lwd=0.8, position=position_dodge(width=0.5)) +

geom_hline(yintercept = 1, linetype = 2) +

geom_hline(yintercept = 1.68, linetype = "dashed", color = "orange", size = 0.8) +

geom_hline(yintercept = 3.47, linetype = "dashed", color = "red", size = 0.8) +

xlab("") +

ylab("Prevalence Ratios (95% CI)") +

geom_errorbar(aes(ymin=Lower, ymax=Upper, col=Group), width=0.5, position=position_dodge(width=0.5)) +

facet_wrap(~Parameter, strip.position="left", ncol=1, scales = "free_y") +

theme(

plot.title = element_text(size = 16, face = "bold"),

axis.text.y = element_blank(),

axis.ticks.y = element_blank(),

axis.text.x = element_text(face = "bold"),

axis.title = element_text(size = 12, face = "bold"),

strip.text.y = element_text(hjust = 0, vjust = 1, angle = 180, face = "bold")

) +

coord_flip() +

theme_bw() +

theme(legend.position = "") +

scale_shape_manual(values = c(15, 17, 15, 17, 15, 17)) +

scale_color_manual(values = c("PSM" = "black", "Non PSM" = "black",

"PSM-nurse" = "#FF4191", "Non PSM-nurse" = "#FF4191",

"PSM-doctor" = "#088395", "Non PSM-doctor" = "#088395"))

dev.off()

sessionInfo()
