# Supplementary material for: The causal effect of workplace violence on mental health and work-related outcomes: a cross-sectional study using propensity score matching
Source: PLoS One. 2025 May 7;20(5):e0321845. doi: 10.1371/journal.pone.0321845 (PMC12058019; doi:10.1371/journal.pone.0321845)
Supplement: Attachment — Submitted filename: Research Article.docx [file pone.0321845.s004.docx]

Research Article

Burnout and Associated Factors among Hospital-Based Nurses in

Northern Uganda: A Cross-Sectional Survey

**Peer Review Comments**

**Identifying the causal effect of workplace violence on depressive symptoms, burnout, sleep problems, work-life balance and intention to quit in Peruvian physicians and nurses: a cross-sectional study using propensity score matching**

**Javier A. Flores-Cohaila1**

**, Brayan Miranda-Chavez2**

**, Cesar Copaja-Corzo3***

| **Sn** | **Comments** |
| --- | --- |
|  | **The authors attempt to identify the causal effect of workplace violence on depressive symptoms, burnout, sleep problems, work-life balance and intention to quit in Peruvian physicians and nurses: a secondary analysis of the 2016 National Healthcare Satisfaction Survey in Peru was conducted** **and Poisson regression models adjusting for all covariates were employed . Consider the following comments below in each section.** |
| **Abstract** | |
| **1** | **Title is too long, it should not exceed more than 14 words. Title should be concise, comprehensive, and clear.** |
| **2** | **The abstract provides a clear overview of the study objectives, and key findings, effectively summarizing the research. Consider design, setting, sample, and tools of study in methods.** |
| **3** | **Abstract should not include citations or abbreviations, if possible. Although its not clear what the design and sampling method for the study.** |
| **6** | **The conclusion emphasizes the need for targeted interventions to address work-place violence in healthcare workforce, aligning with the study findings. Consider elaborating on the specific types of interventions that could be effective in eliminate WV.** |
| **7** | **The keywords are relevant and adequately represent the study focus. However, consider adding additional keywords that capture specific healthcare contexts to improve searchability** |
| **Introduction** | |
| **8** | **The introduction needs more elaborations and a comprehensive background, contextualizing the significance of your study variables. Consider briefly discussing study variables as factors influencing, antecedents, consequences and challenges faced by healthcare system.** |
| **9** | **Consider Integration of Theoretical Frameworks: Consider integrating relevant theoretical frameworks or models of work-place violence (e.g. Justice theory, or Frustration aggression theory) into the discussion to provide a theoretical basis for interpreting the findings and guiding future research and interventions.** |
| **10** | **Add briefly paragraph about significance of the study.** |
| **Materials and Method** | |
| **11** | **Consider including more details about the rationale for choosing a secondary analysis of the 2016 National Healthcare Satisfaction Survey in Peru were selected as the study location. Put research questions.** |
| **12** | **Consider sample size determination** |
| **13** | **The data collection tools and procedures are detailed, including the use of the sociodemographic survey. To enhance reproducibility, consider providing references or additional information about the validity and reliability of these instruments. Also, add years of developing and modifying this tool.** |
| **14** | **Ethical considerations, consider providing more details about how confidentiality was maintained during data collection and analysis.** |
| **Results** | |
| **15** | **Ensure that the tables are properly formatted and labeled for easy interpretation** |
| **Discussion** | |
| **17** | **Secondary analysis research should include a discussion of results. Restate the main research questions that guided the analysis then connect results to existing theories or literatures. Show your point of view and results of your study in discussion. Compare with previous researches.** |
| **18** | **Consider exploring potential contextual factors or methodological limitations that may have influenced these results, providing a more understanding of the findings.** |
| **19** | **However, it could benefit from expanding on the practical implications of the**  **findings for healthcare organizations and policymakers in the region. Specifically,**  **discussing specific strategies or interventions that could be implemented to address the**  **identified factors contributing to transition shock would provide actionable insights for stakeholders.** |
| **References** | |
| **20** | **Use Vancouver style for writing references and in text citation in study. Also, consider language editing.** |

**Thanks for inviting me as a peer reviewer in this journal.**

**Dr. Noura Mohamed Fadl**

***Lecturer of Nursing Administration Department, Faculty of Nursing, Alexandria University***
