# Supplementary material for: The causal effect of workplace violence on mental health and work-related outcomes: a cross-sectional study using propensity score matching
Source: PLoS One. 2025 May 7;20(5):e0321845. doi: 10.1371/journal.pone.0321845 (PMC12058019; doi:10.1371/journal.pone.0321845)
Supplement: Attachment — Submitted filename: [003]Response to Reviewers.docx [file pone.0321845.s006.docx]

**Response letter**

Dear Editor and reviewers, in the following letter, we address each of your comments.

**Reviewer #1**

**Introduction**

*Commentary: Contextualization:* The introduction could better contextualize why workplace violence (WV) is particularly prevalent in Peru. Although it briefly mentions South American perspectives, expanding on cultural and systemic factors unique to Peru (e.g., healthcare infrastructure, socio-political issues) would enhance the relevance.

**Response:** Thank you for this valuable suggestion. We expanded the third paragraph to provide a nuanced overview of significant healthcare system challenges in Peru. Additionally, we refined the transition from the broader Latin American context to the specific case of Peru to strengthen coherence.

*Commentary: Literature Gap:* While the introduction outlines the general impact of WV, it could benefit from citing recent studies that cover broader work environments and settings, helping the reader understand the global context of WV.

**Suggested Studies for Inclusion:**

1. "Mistreatment of nurses by patients and its impact on their caring behaviors: The roles of psychological detachment and supervisor positive gossip"
2. "The role of psychological ownership in linking decent work to nurses' vigor at work"

**Response:** We appreciate these thoughtful recommendations. Although our references already included recent systematic reviews, we decided to enrich the introduction by citing the first study mentioned, as it adds valuable insights into the global context.

**Methods**

*Commentary: Detailed Explanation of Variables:* The manuscript could benefit from a more comprehensive description of the variables, particularly the independent variable (WV). Clarifying the criteria for categorizing different types of WV would strengthen transparency and reproducibility.

**Response:** Thank you for your observation. We expanded our explanation of the categorization process. Specifically, each type of WV was classified based on the original survey items. For example, in the case of "insults," respondents answered the item: *"In the last 12 months, have you been the victim of insults in your workplace? (En los últimos 12 meses, ¿Usted ha sido insultado/a en su lugar de trabajo?)"*

*Commentary: Justification for Using PSM:* Although PSM is a strong methodological choice, explaining why it was chosen over other methods (e.g., instrumental variables) would provide clarity. Additionally, detailing the robustness checks for the PSM model would strengthen confidence in the findings.

**Response:** We appreciate this insightful comment. We added a paragraph to the introduction that outlines the rationale behind choosing PSM, emphasizing its suitability for mitigating selection bias in observational data.

*Commentary: Ethics:* The ethics statement would benefit from a mention of how WV data were anonymized, especially given the sensitive nature of the information.

**Response:** Thank you for this important point. We revised the ethics section to specify that all data in the national survey were anonymized to protect participant confidentiality.

*Commentary: Limitations in Data Collection:* Addressing any limitations in the secondary dataset (e.g., potential biases in the survey questions related to WV) would provide a more balanced perspective.

**Response:** We agree with this observation and have revised the limitations section accordingly. We added: "First, we relied on a secondary dataset not designed to address our specific research questions. Although we adjusted for several confounders, some may remain unaccounted for. Second, WV was self-reported, potentially introducing bias due to social desirability or fear of repercussions."

**Results**

*Commentary: Results Presentation:* While the results are comprehensive, adding summary tables to break down the effects of WV on specific outcomes (e.g., depressive symptoms versus work-life balance) would enhance clarity.

**Response:** Thank you for this suggestion. We added a summary table as appendix material to improve the presentation of the results.

*Commentary: Qualitative Insights:* Given the complex nature of WV, consider adding qualitative insights or hypothetical case examples. For instance, how does experiencing WV potentially influence a healthcare worker’s intention to quit rather than burn out?

**Response:** We fully acknowledge the value of qualitative insights. However, given the quantitative nature of our study, we propose future research that adopts a mixed-methods approach. We added: *"Future studies could incorporate qualitative approaches to capture healthcare workers' experiences, as previous research has suggested that WV is often perceived as 'part of the job.'"*

**Discussion**

*Commentary: Comparative Analysis:* While the discussion addresses global comparisons, it could benefit from a deeper examination of the cultural context in South America and how it influences WV’s prevalence and impact. Comparing the findings with those from other high-risk professions (e.g., police, education) might provide additional insights.

**Response:** We expanded the discussion to contrast the impact of WV on the intention to quit between Peru and Switzerland, illustrating cultural and systemic differences.

*Commentary: Causal Implications:* Although PSM provides some causal inference, the discussion could further emphasize that cross-sectional designs inherently limit causal claims.

**Response:** We agree and have emphasized this limitation in the discussion.

**Suggested Studies for Inclusion:**

1. "Decent work and ethical ideologies of nurses—A multicenter cross-sectional study"
2. "How Decent Work Influences Internal Whistleblowing Intentions in Nurses"

**Response:** We integrated insights from the first suggested study to enrich our discussion on work-life balance and ethical considerations.

**Limitations and Future Research**

*Commentary: Acknowledgment of Cross-Sectional Limitations:* Emphasizing the inherent limitations of cross-sectional designs and the inability to establish true causality would strengthen transparency.

**Response:** We addressed this by adding: *"While our findings portray the effects of WV, longitudinal studies are necessary to capture long-term consequences, such as attrition or migration."*

*Commentary: Further Research:* Suggesting longitudinal studies to track WV’s effects over time would provide a more robust basis for intervention development. Additionally, exploring qualitative studies to understand the lived experiences of healthcare workers dealing with WV could provide richer insights.

**Response:** We included: *"Future research should adopt longitudinal designs to assess the long-term impacts of WV and incorporate qualitative methods to provide deeper insights into healthcare workers' lived experiences."*

**Language and Formatting**

*Commentary: Language:* Several sections of the manuscript would benefit from careful proofreading to enhance readability and eliminate grammatical errors.

**Response:** Thank you. We thoroughly revised the manuscript to improve readability and consistency.

*Commentary: Formatting:* Consistency in terminology (e.g., “workplace violence” versus “WV”) and uniform formatting of statistical terms (e.g., using “95% CI” uniformly) would improve clarity.

**Response:** We reviewed the entire document to ensure uniformity in terminology and statistical reporting.

**Reviewer #2**

**Abstract**

*Commentary 1: Title:* The title is too long; it should not exceed 14 words. The title should be concise, comprehensive, and clear.

**Response:** We revised the title to ensure it is concise and informative.

*Commentary 2: Abstract:* The abstract provides a clear overview of the study objectives and key findings, effectively summarizing the research. Consider design, setting, sample, and tools of study in methods.

**Response:** We appreciate this feedback and have updated the methods section in the abstract to include relevant details.

*Commentary 3: Abstract:* The abstract should not include citations or abbreviations if possible.

**Response:** We removed all abbreviations and citations.

*Commentary 6: Conclusion:* The conclusion emphasizes the need for targeted interventions to address WV in the healthcare workforce. Consider elaborating on the specific types of interventions that could effectively eliminate WV.

**Response:** We expanded the conclusion to describe potential interventions, focusing on organizational policies and training programs.

*Commentary 7: Keywords:* The keywords are relevant and adequately represent the study focus. However, consider adding additional keywords that capture specific healthcare contexts to improve searchability.

**Response:** We added two additional keywords to enhance discoverability.

**Introduction**

*Commentary 8: Background:* The introduction needs more elaboration and a comprehensive background contextualizing the significance of the study variables.

**Response:** We extended the second and third paragraphs to provide an in-depth overview of the key variables and their relevance within the healthcare system.

*Commentary 9: Theoretical Frameworks:* Consider integrating relevant theoretical frameworks or models of workplace violence (e.g., Justice Theory, Frustration-Aggression Theory) into the discussion to provide a theoretical basis.

**Response:** While our study focuses on outcomes rather than theory development, we incorporated relevant theoretical insights to frame the findings more robustly.

*Commentary 10: Significance of the Study:* Add a brief paragraph about the significance of the study.

**Response:** We included a new paragraph highlighting the study’s contribution to the field and its implications for policy and practice.

**Materials and Methods**

*Commentary 11: Rationale for Data Selection:* Include more details about the rationale for choosing the 2016 National Healthcare Satisfaction Survey in Peru.

**Response:** We added: *"This survey was selected due to its extensive scope, robust sampling design, and superior funding compared to primary data collection."*

*Commentary 12: Sample Size Determination:*

**Response:** We included a new section detailing our sample size determination.

*Commentary 13: Data Collection Tools:* Provide references or additional information about the validity and reliability of the instruments.

**Response:** We added citations for validation studies conducted in our country.

*Commentary 14: Ethical Considerations:* Provide more details about maintaining confidentiality during data collection and analysis.

**Response:** We enriched the ethics section with details on data anonymization and secure handling procedures.

**Results**

*Commentary 15: Tables:* Ensure that tables are properly formatted and labeled for easy interpretation.

**Response:** We reviewed and reformatted all tables, ensuring clear presentation.

**Discussion**

*Commentary 17: Discussion of Results:* This section should include a discussion of the results, restating the research questions and connecting the findings to existing theories and literature.

**Response:** We revised the opening of the discussion to restate the research questions and connect our findings to the relevant literature.

*Commentary 18: Contextual Factors:* Expand on potential contextual factors or methodological limitations that may have influenced the results.

**Response:** We believe this is well-covered in the limitations section but added a brief mention in the discussion for emphasis.

*Commentary 19: Practical Implications:* Discuss specific strategies or interventions for addressing WV.

**Response:** We concluded the discussion with a paragraph highlighting practical recommendations for healthcare organizations and policymakers.

**References**

*Commentary 20: Referencing Style:* Use the Vancouver style for writing references and in-text citations.

**Response:** We ensured that all references follow Vancouver style guidelines. Thank you for this observation.

We consider that this new version of the manuscript has improved its quality thanks to your comments.

Kind regards
